# Bioactive VEGF-C from *E. coli*

**DOI:** 10.1038/s41598-022-22960-0

**Published:** 2022-10-28

**Authors:** Khushbu Rauniyar, Soheila Akhondzadeh, Anna Gąciarz, Jaana Künnapuu, Michael Jeltsch

**Affiliations:** 1 Drug Research Program, Faculty of Pharmacy, Biocenter 2, University of Helsinki, P.O.B. 56 (Viikinkaari 5E), 00014 Helsinki, Finland; 2 Individualized Drug Therapy Research Program, University of Helsinki, Helsinki, Finland; 3 Wihuri Research Institute, Helsinki, Finland

**Keywords:** Proteins, Isolation, separation and purification

## Abstract

Vascular endothelial growth factor-C (VEGF-C) stimulates lymphatic vessel growth in transgenic models, via viral gene delivery, and as a recombinant protein. Expressing eukaryotic proteins like VEGF-C in bacterial cells has limitations, as these cells lack specific posttranslational modifications and provisions for disulfide bond formation. However, given the cost and time savings associated with bacterial expression systems, there is considerable value in expressing VEGF-C using bacterial cells. We identified two approaches that result in biologically active *Escherichia coli*-derived VEGF-C. Expectedly, VEGF-C expressed from a truncated cDNA became bioactive after in vitro folding from inclusion bodies. Given that VEGF-C is one of the cysteine-richest growth factors in humans, it was unclear whether known methods to facilitate correct cysteine bond formation allow for the direct expression of bioactive VEGF-C in the cytoplasm. By fusing VEGF-C to maltose-binding protein and expressing these fusions in the redox-modified cytoplasm of the Origami (DE3) strain, we could recover biological activity for deletion mutants lacking the propeptides of VEGF-C. This is the first report of a bioactive VEGF growth factor obtained from *E. coli* cells circumventing in-vitro folding.

## Introduction

Vascular endothelial growth factor VEGF-C is essential for the embryonic development of the lymphatic vasculature and its maintenance in the adult organism^1^. Unlike VEGF-A, which is secreted as an active growth factor, the VEGF-C polypeptide (*unprocessed VEGF-C*) requires two proteolytic cleavages for activation^2^. The first C-terminal cleavage (resulting in *pro-VEGF-C*) occurs constitutively in the oxidizing environment of the endoplasmic reticulum/Golgi apparatus and is mediated by protein convertases^3^. The second cleavage step takes place in the extracellular environment and is mediated by multiple enzymes, including plasmin^4^, ADAMTS3^5^, PSA/KLK3, cathepsin D^6^, and thrombin^7^. It results in N-terminally slightly different mature forms of VEGF-C which differ in their activity towards their receptors VEGFR-2 and VEGFR-3^6^. With about 9% of its amino acid residues being cysteine residues, VEGF-C belongs to the 50 cysteine-richest long (> 400 aa) proteins coded by the human genome (https://github.com/mjeltsch/cysteine). With the increasing number of cysteines, the difficulty of correct disulfide bond formation increases exponentially. Moreover, the high number of genes in most animal genomes for enzymes catalyzing disulfide bond formation and isomerization supports the idea, that VEGF-C might be difficult to produce in a prokaryotic expression host like *E. coli*. In addition, it is known that eukaryotic chaperones are known to aid in the correct folding of the closely related VEGF-A^8,9^.

To produce VEGF-A, PlGF, and VEGF-B for crystallization purposes, solubilization from inclusion bodies and subsequent refolding under the appropriate conditions have been deployed^10–15^. However, the refolding efficacies were never explicitly reported. Final yields of active protein have been reported to be in the range of 2–4 mg/L of bacterial culture^15^ or 10 g bacterial wet weight^10^, which translates to estimated refolding efficiencies below 1%. Contrary to the above proteins, all reported structures for VEGF-C and VEGF-D were solved using proteins from eukaryotic expression systems^16,17^.

There are two principal methods to promote the correct folding of cystine-containing proteins in the cytoplasm of *E. coli*: (1) by modulating the intracellular redox environment^18^ as done in *E. coli* strains such as Origami, AD494 (both Novagen), or SHuffle (New England Biolabs), and (2) by co-expressing enzymes catalyzing disulfide bond formation and isomerization (as e.g. done in the CyDisCo system^19,20^), reviewed by de Marco^21^. Alternatively, the oxidizing environment of the periplasm^22^ or extracellular space^23^ can be utilized. Also, combinations of these strategies have been used^24^. In this study, we tested four different approaches: (1) CyDisCo, (2) periplasmic targeting, (3) expression in a redox-modulated cytoplasm, and (4) in vitro folding of solubilized inclusion bodies. The latter two approaches were successful. Expression in a redox-modulated cytoplasm was first efficiently achieved by MBP-tagging VEGF-C, but we also were able to recover low levels of biological activity from non-MBP-tagged VEGF-C.

## Results

### The cysteine-rich C-terminal domain of VEGF-C appears to be responsible for the slow secretion of VEGF-C

**Figure 1:**
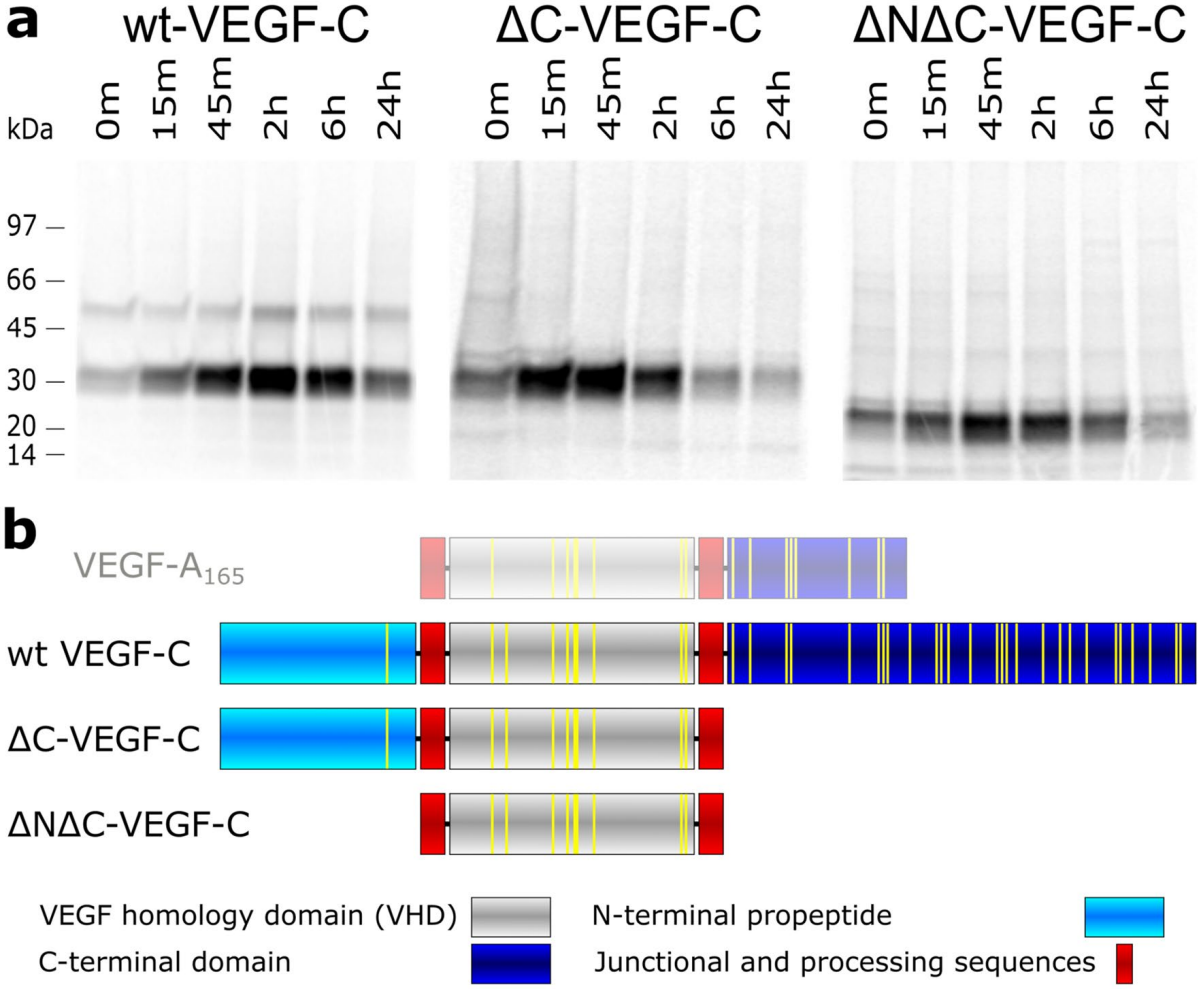
The C-terminal domain of VEGF-C slows down biosynthesis and secretion of VEGF-C. (**a**) Transfected 293T cells were pulsed with S^35^-labeled Met/Cys and chased for the indicated amounts of time. Maximum levels of VEGF-C were detected after 2 h for the wild-type cDNA. When the C-terminal domain was deleted, the maximum protein levels were seen in the supernatant already after 15–45 min. Additional deletion of the N-terminal domain did not accelerate biosynthesis and secretion further but rather slowed it down a bit. (**b**) A schematic of the domain structures of the VEGF-C proteins is shown including a comparison with VEGF-A_165_. Cysteine residues are depicted by yellow lines.

If correct disulfide bond formation represented a bottleneck in the production of VEGF-C, one would expect that deleting the cysteine-rich C-terminal domain accelerates the folding and thus the biosynthesis and secretion of VEGF-C. We found that deleting the C-terminal domain (ΔC) as well as deleting both the N-terminal propeptide and the C-terminal domain (ΔNΔC) speeded up the secretion of VEGF-C. In a pulse-chase experiment, the maximal levels of VEGF-C in the supernatant were measured for the wild-type VEGF-C at 2 h, the ΔC mutant between 15 and 45 min, and the ΔNΔC mutant at 45 min (Fig. 1).

### Partial lack of N-linked glycosylation does not preclude VEGF-C binding to VEGFR-3

**Figure 2:**
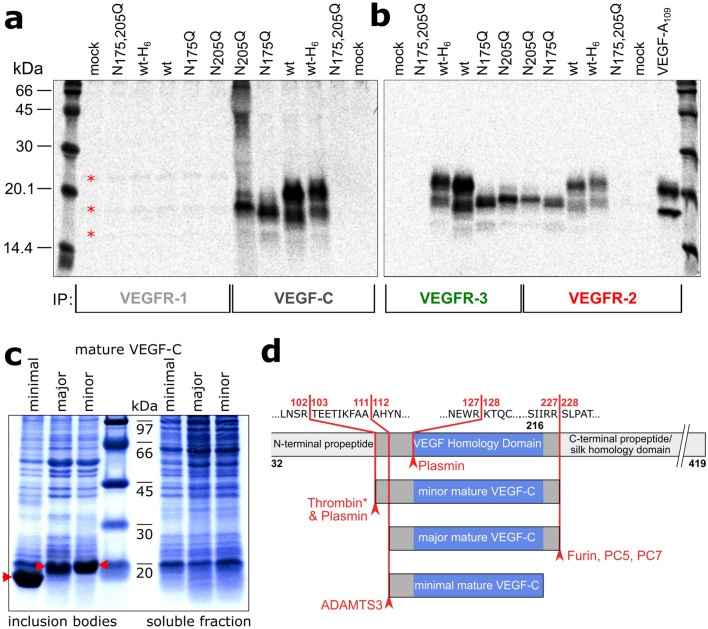
Single glycosylation mutants of mature VEGF-C expressed in 293T cells can bind VEGFR-2 and VEGFR-3, but mature VEGF-C aggregates into inclusion bodies when expressed in *E. coli* BL21 (DE3). (**a**, **b**) Immunoprecipitation (IP) of [^35^S]-labeled VEGF-C from media of 293T cells transfected with the indicated single and double glycosylation mutants of VEGF-C. The equivalent N > W glycosylation mutants gave identical results (Supplementary Fig. S1). Note that when VEGFR-1 binding is studied, the isoforms of VEGF-A endogenously expressed by 293T cell result in 3 distinct faint bands (marked by asterisks). (**c**) Coomassie-stained PAGE gel of the cytoplasmic inclusion body fraction and the cytoplasmic soluble fraction. None of the inclusion body fractions or the soluble fractions show any biological activity. The expected sizes for the minimal, major, and minor mature VEGF-C are ~ 11.8 kDa, ~ 13.2 kDa, and ~ 14.2 kDa, respectively, which are denoted by red arrows. (**d**) Schematic of the truncation mutants and how they relate to the naturally occurring proteolytically generated mature forms of VEGF-C.

A major limitation of bacterial hosts for the expression of secreted eukaryotic proteins is the lack of glycosylation. Because the VEGF homology domain (VHD) of VEGF-C features two N-linked glycosylation sites at amino acids 175 and 205, we investigated whether the glycosylation of VEGF-C is a requirement for receptor binding. Hence, we produced single and double glycosylation mutants of VEGF-C by replacing one or both Asn residues with Gln and Trp, respectively, resulting in six different glycosylation mutants designated N_175_Q, N_205_Q, and N_175,205_Q, and N_175_W, N_205_W, and N_175,205_W. Replacement by glutamine was chosen to maintain the chemical nature of the asparagine residue and tryptophan to mimic the bulky carbohydrate moiety. When expressed in 293T cells, all single mutants were able to bind VEGFR-2 and VEGFR-3, indicating that no specific interaction of any single carbohydrate chains is needed for receptor binding. However, the complete absence of glycosylation resulted in failure to be expressed (supplemental Figure S1) and secreted (Fig. 2a, b, supplemental Figure S1), similar to other secreted proteins^25^.

### VEGF-C expressed in the cytoplasm of *E. coli* BL21 (DE3) is found exclusively in inclusion bodies

Based on the glycosylation data, we reasoned that glycosylation would not be a per-se requirement for receptor binding or activation and we proceeded to produce VEGF-C in *E. coli*. We designed three different truncated cDNAs devoid of the C-terminal and N-terminal domains. These forms corresponded to the major and minor mature forms of VEGF-C as produced by 293 cells when transfected with full-length VEGF-C cDNA^2^, and to a minimal form with the same N-terminus as the major mature form of VEGF-C, but which lacked the C-terminal sequences required for neuropilin-2 interaction (Fig. 2c, d)^26^. When these three forms were produced in the cytoplasm of *E. coli* BL21 (DE3), all of the protein did aggregate in inclusion bodies (Fig. 2c), and no biological activity could be seen in the Ba/F3-VEGFR-3/EpoR assay in the soluble fraction (data not shown). Based on this observation and the suspected difficulty of disulfide bond formation, we decided to apply CyDisCo, which is a system enhancing soluble expression of disulfide-bonded proteins in the cytoplasm of *E. coli*.

### Coexpression with a sulfhydryl oxidase and disulfide bond isomerase (CyDisCo system) does not yield active VEGF-C

Previously, we had unsuccessfully tried to express active VEGF-C in the cytoplasm of *E. coli* strain AD494 (DE3), which has a mutation in the thioredoxin reductase B gene, which is one of the key players in the pathways reducing disulfide bonds in the cytoplasm of *E. coli*^27^ (data not shown). The more recently developed, enhanced intracellular *E. coli* expression system CyDisCo differs from AD494 and similar *E. coli* strains in that it does not modify the cytoplasmic redox environment, but only facilitates correct disulfide bonding by co-expressing enzymes catalyzing disulfide bond formation (a sulfhydryl oxidase such as Erv1p) and isomerization (human PDI)^19,20,28^. CyDisCo system proved efficient for soluble expression of a variety of disulfide-bonded proteins including antibody fragments^28^, BPTI, vtPA, resistin, Ero1α, CFS3, BMP4, the catalytic light chain of enterokinase, IFNα 2, IL-17^20^, alkaline phosphatase, AppA^19^ and many others. Using this strategy, we expressed different VEGF-C constructs including truncation mutants and the full-length protein (Phe32-Ser419). However, when we analyzed the cytoplasmic fraction of *E. coli* transformed with these constructs, none showed any biological activity towards VEGFR-3 (Supplementary Fig. S2). Similar to our first attempts, the VEGF-C proteins aggregated into inclusion bodies visible as thick bands in the total cell protein fractions on SDS-PAGE gels (Supplementary Fig. S2).

### Periplasmic expression

Because of the unsuccessful results using the CyDisCo system, we turned to periplasmic targeting. Targeting a protein to the periplasmic space, which is a more favorable environment for disulfide bridge formation, has been used to produce active extracellular proteins without the need for additional folding. However, this approach has been most successful with small proteins containing relatively few disulfide bonds, such as IL-3 or IL-4^29,30^. We wanted to know whether a similar approach would succeed for a protein like VEGF-C, whose cystine content well exceeds the average for cystine-knot growth factors. We inserted sequences for the pelB signal peptide in front of 4 different cDNAs coding for different forms of mature and full-length VEGF-C. After transformation of these constructs to BL21 (DE3) *E. coli*, we assayed the periplasmic protein fraction for VEGF-C protein and activity but none of the periplasmic extracts showed any significant biological activity in the Ba/F3-VEGFR-3/EpoR assay (Supplementary Fig. S3).

### Using maltose-binding protein as a solubility tag for VEGF-C yields active, mature VEGF-C in *E. coli* Origami (DE3) cytoplasm

**Figure 3:**
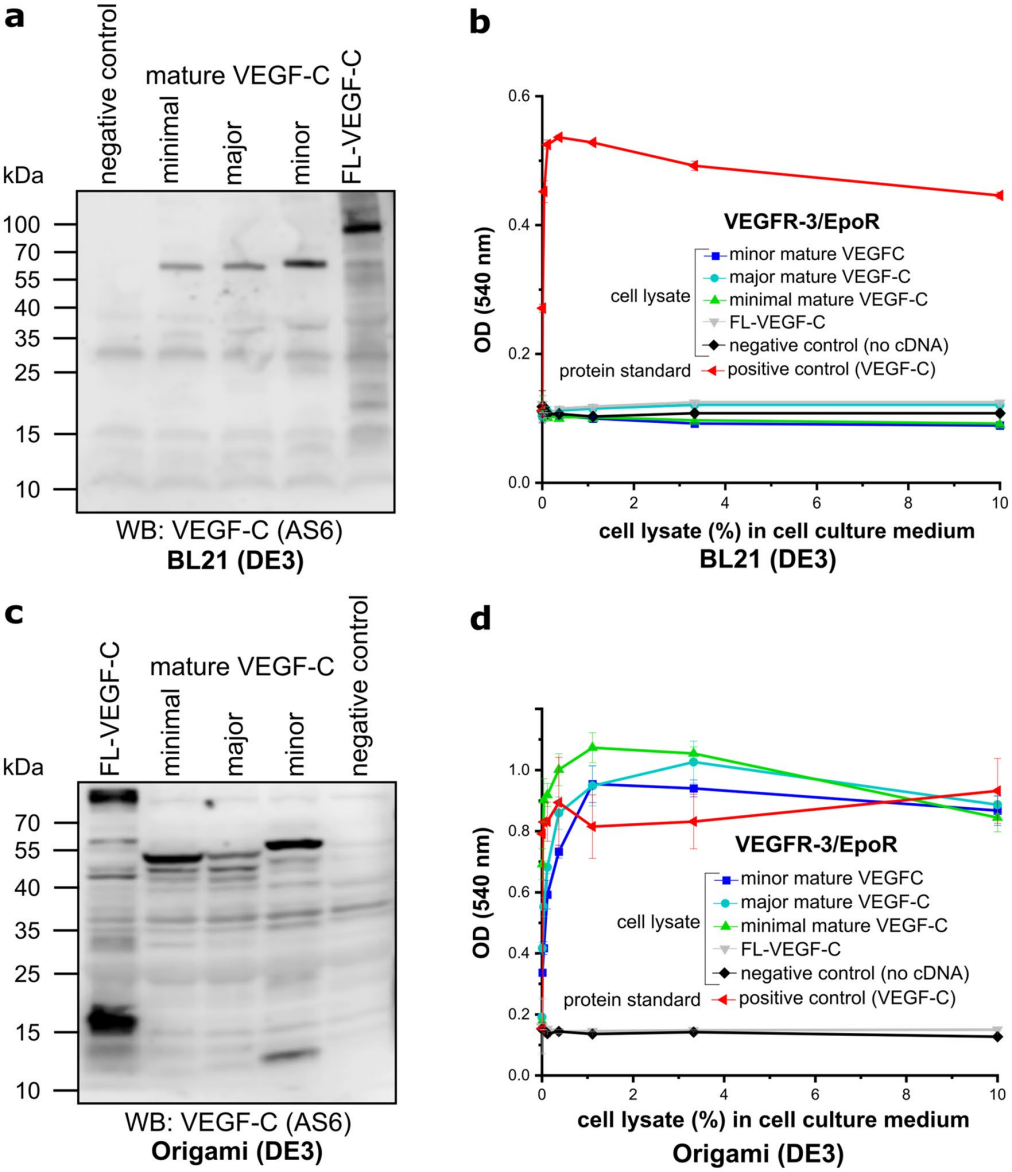
MBP-tagged VEGF-C is active when expressed in the cytoplasm of *E. coli* Origami (DE3) but inactive in BL21 (DE3) strain. (**a**, **b**) *E. coli* strain BL21 (DE3)-produced MBP-tagged mature VEGF-C is inactive, but largely protected from proteolytic degradation due to inclusion body formation. (**c**, **d**) The cytoplasm of *E. coli* strain Origami (DE3) transformed with the same constructs contains biologically active VEGF-C, but the Western blot shows that the MBP-tagged VEGF-C is subject to heterogenous proteolytic processing. In neither of the two *E. coli* strains did transformation with MBP-tagged full-length VEGF-C cDNA result in biologically active VEGF-C. The expected sizes for the MBP-tagged minimal, major, minor mature VEGF-C and full-length VEGF-C are ~ 56.9 kDa, ~ 58.3 kDa, ~ 59.5 kDa, and ~ 89 kDa, respectively. The MBP-tag accounts for ~ 40 kDa. (n = 3) Error bars indicate ± SD.

In our previous cytoplasmic expression attempts, VEGF-C aggregated into inclusion bodies independently of whether it was expressed in a redox-modified (strain AD494 with mutation in thioredoxin reductase (ΔtrxB)) or non-redox-modified (in the presence of CyDisCo system) host. Searching for alternative means to prevent the formation of inclusion bodies, we expressed VEGF-C as a fusion protein with maltose-binding protein (MBP), which is known for its ability to enhance the solubility of its fusion partners^31^. We expressed four different forms of MBP-tagged VEGF-C in *E. coli* strain BL21 (DE3). Although VEGF-C was visible in the soluble cytosolic fraction on SDS-PAGE (Fig. 3a), no activity was observed when testing cytoplasmic extracts for biological activity (Fig. 3b). However, expression of the same MBP-VEGF-C constructs in the cytoplasm of *E. coli* strain such as Origami (DE3) (mutation in both glutathione reductase (Δgor) and ΔtrxB) facilitated correct disulfide bridge formation and folding of VEGF-C. The cytosolic fraction of the *E. coli* strain Origami (DE3) expressing the mature forms of MBP-tagged VEGF-C showed activity in the Ba/F3-VEGFR-3/EpoR assay (Fig. 3c, d).

**Figure 4:**
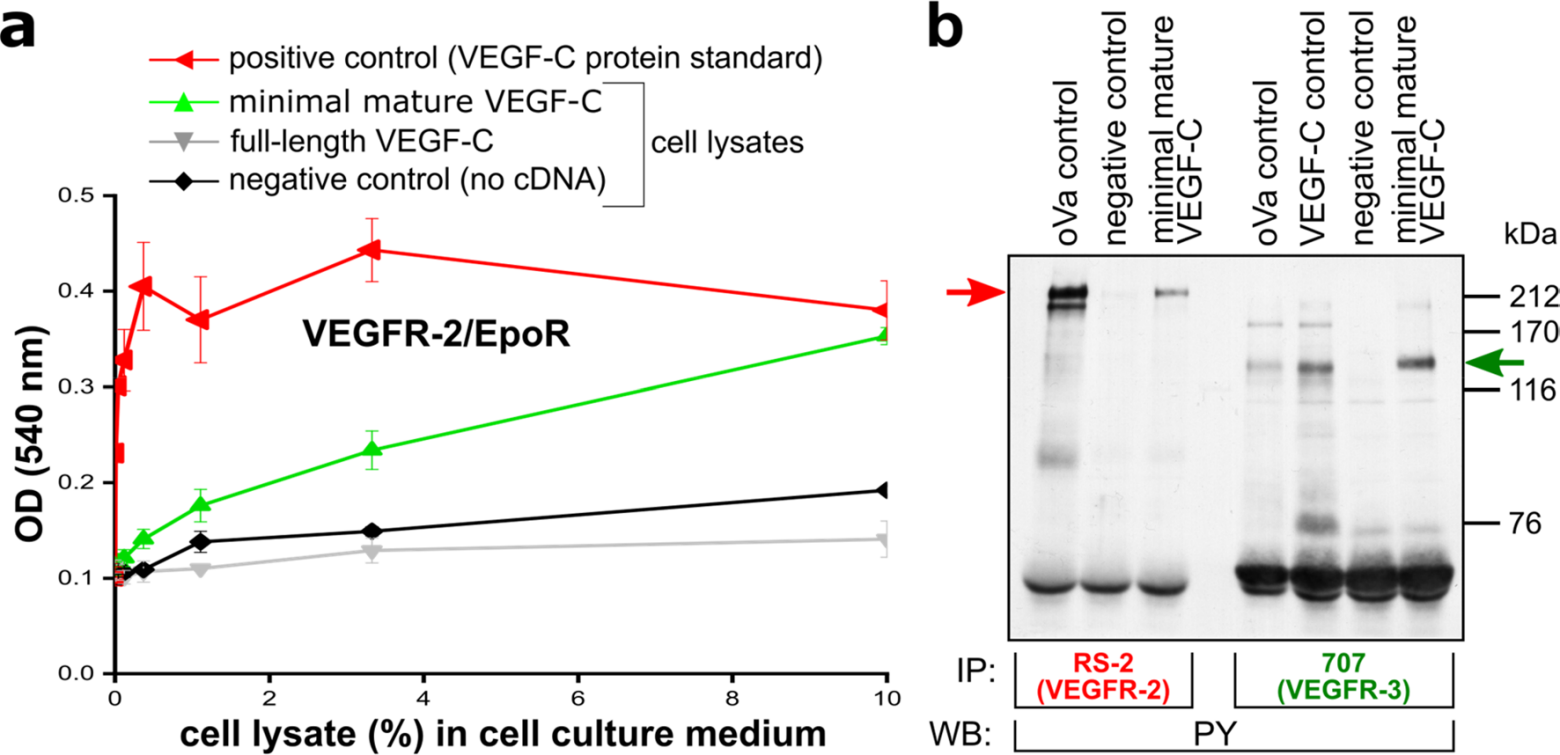
MBP-tagged minimal mature VEGF-C expressed in the cytoplasm of *E. coli* Origami (DE3) can activate both VEGFR-2 and VEGFR-3. Minimal mature MBP-VEGF-C expressed in Origami (DE3) (**a**) promotes the survival of Ba/F3 cells stably transfected with a VEGFR-2/EpoR chimeric receptor, (n = 2) Error bars indicate ± SD, and (**b**) stimulates the phosphorylation of VEGFR-2 and VEGFR-3 expressed by PAE cells without the need of refolding. PY denotes phosphorylated tyrosine. The major phosphorylated receptor bands are indicated by arrows.

The minimal form of mature VEGF-C showed the strongest response in the Ba/F3-VEGFR-3/EpoR assay and was used for most subsequent assays. We confirmed its biological activity also for the alternative VEGF-C receptor (VEGFR-2) in a bioassay using Ba/F3 cells expressing VEGFR-2/EpoR chimeric receptors (Fig. 4a). This minimal form of mature VEGF-C also successfully stimulated the phosphorylation of VEGFR-2 and VEGFR-3 stably expressed by PAE cells (Fig. 4b).

### Characterization of the MBP-tagged VEGF-C

**Figure 5:**
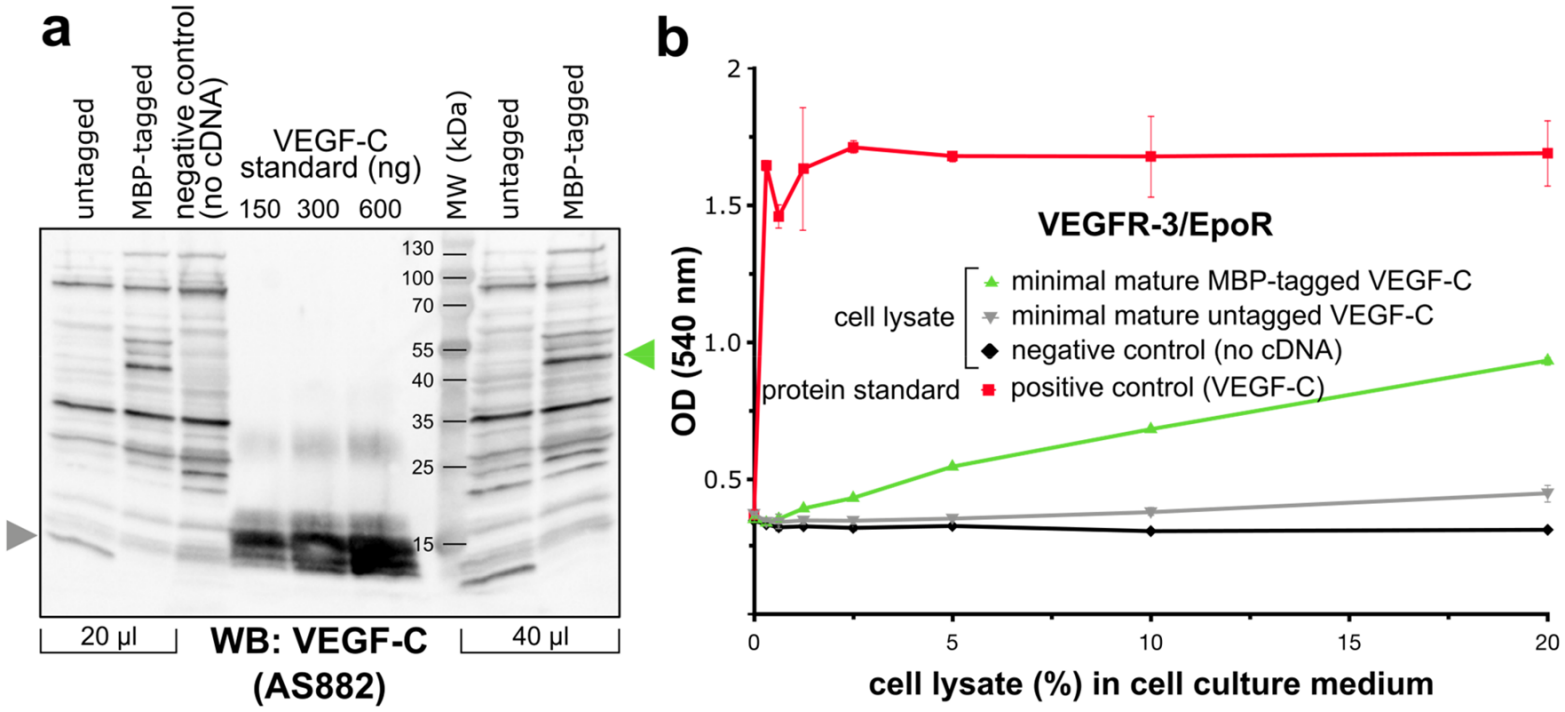
VEGF-C expression with the fusion tag MBP increases significantly the bioactivity of VEGF-C produced in the cytoplasm of *E. coli* Origami (DE3). (**a**) Western blot comparing MBP-tagged and untagged VEGF-C. The MBP-tagged and untagged VEGF-C bands are indicated by green and gray arrows, respectively. (**b**) The untagged VEGF-C exhibits only very low levels of bioactivity in Ba/F3-VEGFR-3/EpoR assay. (n = 2) Error bars indicate ± SD.

To confirm that the MBP-tag combined with Origami (DE3) strain was responsible for the gain in biological activity, we subcloned the coding sequence for minimal mature VEGF-C from the kanamycin resistance plasmid pET28b(+) to the equivalent ampicillin resistance plasmid pET15b(+) to be able to transform it into the kanamycin-resistant Origami (DE3) cells. To our surprise, also the untagged minimal mature VEGF-C expressed in Origami (DE3) cells showed biological activity in the Ba/F3-VEGFR-3/EpoR assay (Supplementary Fig. S4). However, when we compared the amounts of MBP-tagged and untagged minimal mature VEGF-C from a quantified Western blot, the activity of the untagged protein remained much below the activity of the MBP-tagged protein (Fig. 5).

**Figure 6:**
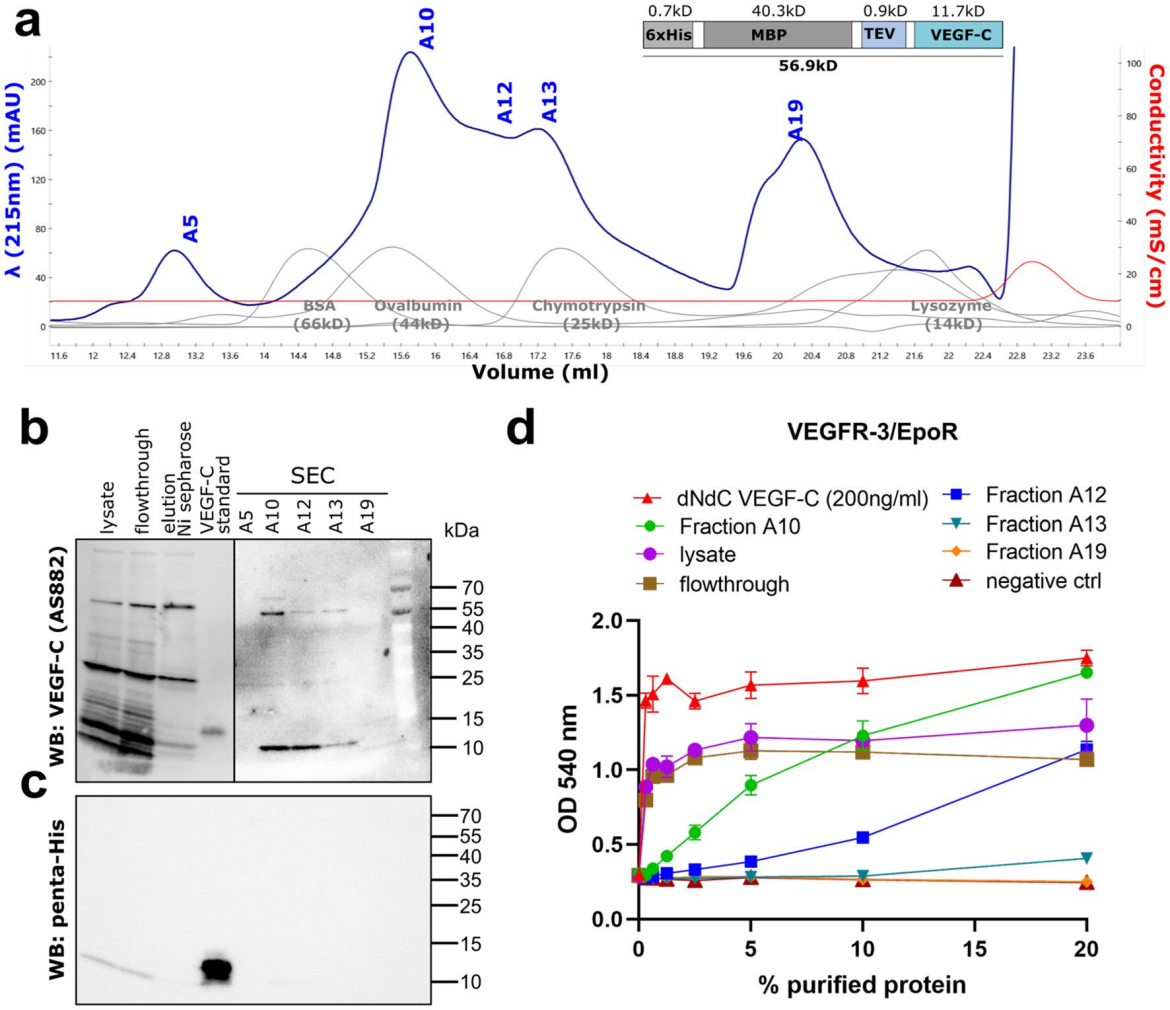
Purification of MBP-tagged VEGF-C using Ni sepharose affinity chromatography. (**a**) Size-exclusion chromatogram of the MBP-VEGF-C fusion protein. (**b**) The fusion protein could be purified using Ni sepharose affinity chromatography despite the absence of the hexahistidine tag, which appeared to be cleaved off by *E. coli*-derived proteases (**c**). A variable amount of the MBP-VEGF-C was also cleaved between the VEGF-C and the MBP moiety. This resulting ~ 56.9 kDa band represents the MBP-VEGF-C fusion protein, while the ~ 11.7 kDa band is VEGF-C alone. These forms cannot be separated by size exclusion chromatography because a single VEGF-C dimer can contain one MBP-VEGF-C fusion monomer and one VEGF-C monomer without the MBP moiety. 100 ng of a hexahistidine-tagged VEGF-C standard^26^ was used as a reference for the Western blot. Note that the right side of the blot (**a**) has longer exposure to show the weaker bands. (**d**) Ba/F3-VEGFR-3/EpoR assay showing the activity of purified fractions, lysate and flowthrough. Similar activity in lysate and flowthrough indicates that majority of the active VEGF-C remained in the flowthrough. The line graph was generated using GraphPad Prism version 8.2.4 for Windows, GraphPad Software, San Diego, California USA, www.graphpad.com (n = 2) Error bars indicate ± SD.

Further, we purified the MBP-tagged minimal mature VEGF-C by immobilized metal chelate affinity chromatography using Ni sepharose followed by size exclusion chromatography (SEC) (Fig. 6a). We analyzed the purified fractions by SDS-PAGE/Western blotting (Fig. 6b, c) and tested them in the Ba/F3-VEGFR-3/EpoR assay (Fig. 6d). The majority of biological activity was recovered from fraction A10 corresponding to a size of approximately 40 kDa.

**Figure 7:**
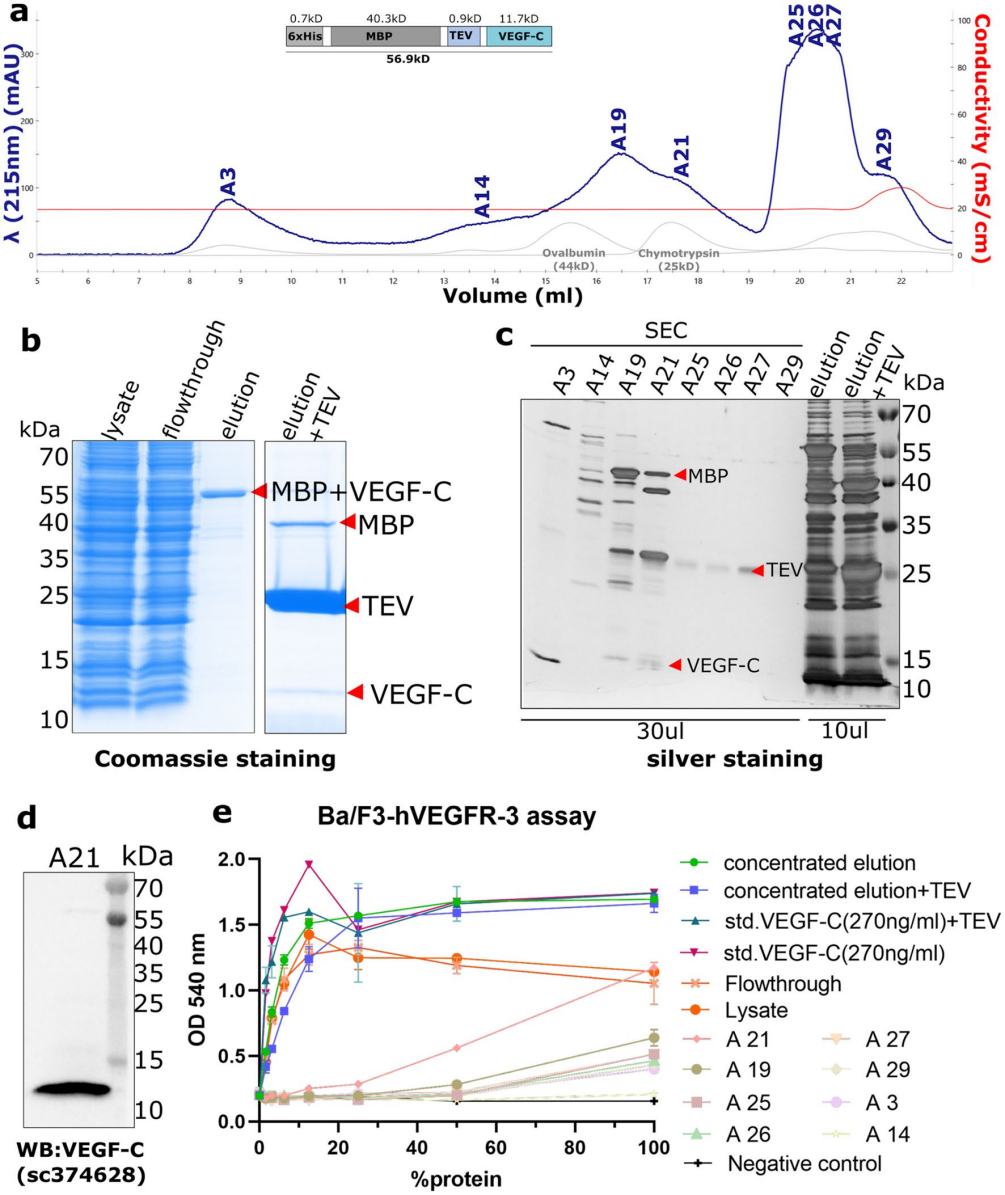
Purification of MBP-tagged VEGF-C using Amylose affinity chromatography. (**a**) Size-exclusion chromatogram of the MBP-VEGF-C fusion protein. (**b**) The fusion protein could be purified using Amylose affinity chromatography and the MBP tag could be cleaved using TEV protease. The peak fractions from SEC were resolved in SDS-PAGE followed by (**c**) silver staining and (**d**) western blotting. The bands corresponding to MBP + VEGF-C, MBP, TEV, and VEGF-C are indicated by red arrows. (**e**) Ba/F3-VEGFR-3/EpoR assay showing the activity of elution (with/without TEV cleavage), purified fractions, lysate, and flowthrough. Similar activity in lysate and flowthrough indicates that the majority of the active VEGF-C remained in the flowthrough. The line graph was generated using GraphPad Prism version 8.2.4 for Windows, GraphPad Software, San Diego, California USA, www.graphpad.com (n = 2) Error bars indicate ± SD.

Since the majority of the active VEGF-C remained unpurified in the flowthrough of the Ni sepharose affinity chromatography, we further used amylose affinity chromatography followed by cleavage of the MBP tag using TEV protease and then SEC to purify the active VEGF-C (Fig. 7). The bioactivity of the purified fractions was analyzed using Ba/F3-VEGFR-3/EpoR assay, and the majority of bioactivity was contained in fraction A21. Similar level of bioactivity from both MBP-cleaved VEGF-C and uncleaved fusion protein indicated that removal of the MBP tag is not required to recover biological activity of VEGF-C.

### No activation of bacterial full-length VEGF-C by the 293T cell secretome

Unsurprisingly, the full-length VEGF-C cDNA, when used for protein production in *E. coli*, did not yield active protein, because unprocessed, full-length VEGF-C does not activate VEGFR-3^5^, and the specific proteins needed for its activation are not produced by *E. coli*. Hence, we exposed the bacterial full-length protein to 293T cells, which secrete ADAMTS3 and CCBE1, both of which are sufficient for the activation of pro-VEGF-C^5^. However, this procedure did not increase the biological activity of the full-length VEGF-C as measured by the Ba/F3-VEGFR-3 assay (Supplementary Fig. S5).

### Active, mature VEGF-C can also be obtained by folding solubilized inclusion bodies

While getting the first disappointing results in our attempts to directly generate bioactive VEGF-C in the *E. coli* cyto- or periplasm, we started to evaluate different folding conditions for solubilized inclusion bodies. To obtain biologically active protein, we solubilized the inclusion bodies of the minimal mature form of VEGF-C produced from a truncated, synthetic cDNA using 6M guanidinium chloride (GuHCl) and performed four successive limited screens to identify and optimize suitable folding conditions (see Table S3 for the exact folding conditions). The most efficient folding was obtained under slightly alkaline conditions with high chaotrope and salt concentrations, using reduced/oxidized glutathione as a redox pair (buffer composition: 100 mM Tris pH 8.5, 2 mM EDTA, 550 mM GuHCl, 264 mM NaCl, 11 mM KCl, 10 mM GSH/0.1 mM GSSG).

**Figure 8:**
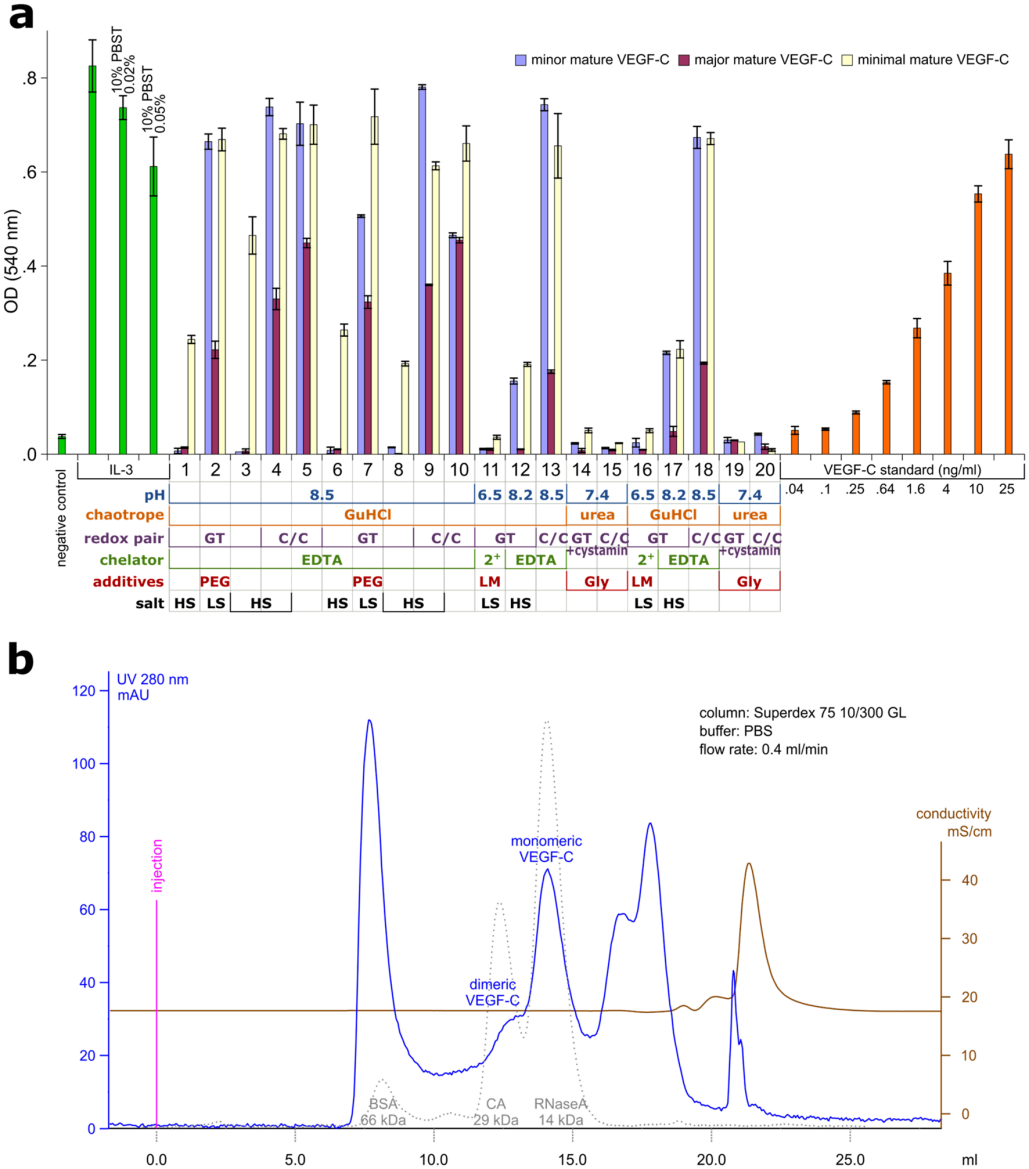
Ba/F3-hVEGFR-3/EpoR bioassay and size-exclusion chromatogram of refolded VEGF-C inclusion bodies (fourth/final screen). (**a**) The Ba/F3-hVEGFR-3/EpoR cell line requires supplementation with either IL-3 or VEGF-C for survival, which was measured 48 h after adding the refolding samples at a concentration of 10% by the ability to metabolize 3-(4,5-dimethylthiazol-2-yl)-2,5-diphenyltetrazolium bromide (MTT). Three different forms of mature VEGF-C were analyzed and compared to a VEGF-C protein standard produced in insect cells^26^. *GuHCl* guanidine hydrochloride, *GT* glutathione, *C/C* cysteine/cystine, *EDTA* ethylenediaminetetraacetic acid, *2* + Mg2+/Ca2+ 2.2 mM, *PEG* polyethylene glycol, *Gly* glycine, *LM* lauryl maltoside, *HS* high salt, *LS* low salt. (n = 2) Error bars indicate ± SD. (**b**) After removing material that had been precipitating during the refolding, the sample was resolved by size exclusion chromatography on a Superdex 75 10/300 GL column (GE Healthcare). The active, dimeric peak represented only a minor fraction of the total protein.

We further determined that these conditions were also suitable to obtain active mature VEGF-C from truncated cDNAs coding for the naturally occurring major and minor mature forms of VEGF-C^32^. However, there were clear differences in the ability to fold between these three forms. The minor mature form and the minimal receptor binding domain both appeared to fold much easier compared to the major, mature form. A quantitative comparison of the protein activity (Fig. 8a) with a VEGF-C protein standard produced in insect cells^26^ showed that the yield was approximately 0.01 mg per liter of *E. coli* culture, and that only about 0.8‰ of the total bacterial VEGF-C protein could be recovered as biologically active VEGF-C using this refolding and purification scheme (Supplementary Fig. S6). Because of the high purity of the inclusion body preparation (estimated > 70%, see Fig. 2a), and because the majority of misfolded protein precipitated during the dialysis step after the refolding, the active VEGF-C protein could be easily purified in a single step using size exclusion chromatography (Fig. 8b).

## Discussion

Recombinant proteins produced in *E. coli* lack important post-translational modifications of mammalian proteins (most notably glycosylation of secreted proteins). However, using *E. coli* as an expression host is simpler and often more productive compared to mammalian cells. For its low costs, production in *E. coli* is the method of choice if the downstream applications do not require eukaryotic-specific posttranslational modifications. Also in the manufacturing of biologicals, *E. coli* is sometimes used (e.g., for insulin^33^). However, not all mammalian proteins do fold correctly in *E. coli*. Cystine knot proteins, which are virtually unknown in the bacterial kingdom, but have developed in fungi, plants, and animals, are often problematic, and the production of extracellular cystine knot proteins in the *E. coli* cytoplasm leads mostly to protein aggregation and the formation of inclusion bodies^21^.

The vascular endothelial growth factor (VEGF) family of growth factors is interesting because its members are targets for inhibition, e.g. of tumor-induced blood vessel growth^34–37^, and promotion, e.g. of lymphatic vessel growth in lymphedema^38^. Inhibition has been mostly achieved by antibodies, which are still almost exclusively produced in mammalian cells for both research and therapeutic purposes, and successful attempts to produce full antibodies in *E. coli* are sparse^39^. However, most members of the VEGF growth factors have been produced successfully in *E. coli*^10,11,40–43^ but the exact refolding conditions have not been reported in some of these works^12,15^. Notably, the successful production of the lymphangiogenic VEGFs (VEGF-C and VEGF-D) has not been reported in *E. coli* to date. The protein production for structural and functional studies on VEGF-C and VEGF-D has been performed for the last 20 years in insect or mammalian cells^6,44–47^. Some commercial suppliers offer VEGF-C produced in *E. coli* from a truncated cDNA, but the reported biological activities are significantly lower compared to corresponding proteins produced by mammalian hosts^48^. When VEGF-C expression is performed from a truncated cDNA, an odd 9th cysteine in the VEGF homology domain interferes with correct disulfide bond formation^17,49^. This cysteine is present in both VEGF-C and VEGF-D but absent from all other VEGFs.

Counter-intuitively, commercially available growth media for lymphatic endothelial cells (LECs) are mostly not supplemented with the lymphangiogenic VEGF-C, but with VEGF-A, which is the primary growth factor for blood vascular endothelial cells (BECs). Unlike VEGF-C, VEGF-A cannot activate the primary lymphangiogenic growth factor receptor VEGFR-3^1,50^. Hence, a readily available source of biologically active VEGF-C for lymphatic endothelial cell culture is of interest.

We noticed that in mammalian cells, producing VEGF-C from a full-length cDNA is slower compared to a truncated cDNA, most likely due to the vastly increased possibilities for disulfide bond formation. But irrespective of whether we used a full-length or a truncated cDNA, our initial attempts to produce VEGF-C in bacteria led to inclusion body formation, a typical result for many other cystine knot growth factors^51–53^. In eukaryotic cells, protein disulfide isomerases (PDIs) are deployed to ensure rapid isomerization into the correct bonding pattern. While many secreted proteins with disulfide bonds have been successfully co-expressed in *E. coli* with the CyDisCo expression system, we failed to generate bioactive VEGF-C with this strategy.

However, even in the Kringle-2 serine protease fragment vtPA^20,54^, which is used as the acid test for disulfide bond formation in *E. coli*, only 5.1% of the amino acid residues are cysteines, while in the various mature VEGF-C forms the cysteine content is between 7.2 and 8.6%. From the 18 cysteine residues in the dimer, two remain unpaired^16,49,55^, and correct inter- and intramolecular disulfide bond formation in VEGF-C is likely a rare event compared to aggregation, even with PDI assistance. A large number of different PDI family members (21, and an even larger number of thioredoxin domain-containing proteins) suggests that specialization has occurred among these proteins. Possibly, with *S. cerevisiae* ERV1 and *H. sapiens* PDI of the CyDisCo system, we simply tested the wrong enzymes. The same might have been the case in a previous attempt to improve the quality of VEGF-C by co-expressing CALR, CDC37, PH4B, PDIA3, or PPIB in insect cells^56^. It is also possible that VEGF-C folding benefits from the assistance by a specialized chaperone, as was reported for the closely related VEGF-A^57^. However, even though the redox environment of the bacterial cytoplasm does not per se prevent the formation of disulfide bonds, the typical environment of most PDIs is the eukaryotic endoplasmic reticulum, which features a different redox environment compared to the cytoplasm^58^.

We chose two parallel strategies to produce bioactive VEGF-C: Folding from solubilized inclusion bodies and preventing the formation of inclusion bodies by fusing VEGF-C to the solubility-enhancing maltose-binding protein^31^. The first approach led to the identification of suitable folding conditions, which were similar to conditions previously reported to work for other VEGF family members.

Fusing VEGF-C to the solubility-enhancing MBP was unsuccessful on its own in the *E. coli* strain BL21, but when combined with the modified cytoplasmic redox-environment of the Origami *E. coli* strain led to the generation of bioactive VEGF-C. Compared to the soluble VEGF-C in the Origami strain, the insoluble, aggregated VEGF-C in the BL21 strain was largely protected from proteolytic degradation. Proteolytic degradation is at least partly responsible for the low yield of VEGF-C after purification (compare Fig. 3a with Fig. 3c, Figs. 6 and 7). The Western blotting with pentahis-antibody detection indicates that proteolytic processing removes the hexahistidine tag from the MBP-VEGF-C fusion protein. However, VEGF-C binds nevertheless to the Ni sepharose similar to what has been shown previously for VEGF-A^59^. Yet, this binding is not efficient, and majority of active VEGF-C remains in the flowthrough during this step (Fig. 6b–d). VEGF-C recovery could be increased to 0.6 mg/L of bacterial culture using amylose resin, which binds to the MBP tag. However, due to proteolytic processing (see Fig. 7c, e) and/or improper folding of the MBP tag in the Origami strain, a large amount of the active VEGF-C was still present in the flowthrough.

We were surprised to see that bioactive VEGF-C could also be produced in the *E. coli* Origami strain without the need for the MBP moiety. However, a side-by-side comparison of quantified proteins showed that without the MBP moiety, the activities that we could recover were minimal compared to the MBP-tagged VEGF-C. Such activities were even sometimes undetectable in our experiments depending on the sensitivity of the bioassay. Interestingly, after Ni sepharose affinity chromatography, the major biologic activity was recovered from the fraction containing VEGF-C fused with a partially proteolytically processed MBP. In addition, MBP-tagged VEGF-C eluted from the amylose resin had a very similar level of bioactivity in Ba/F3-VEGFR-3 assay compared to VEGF-C, from which the MBP moeity had been removed by TEV cleavage. This indicates that removal of the MBP tag is not required to recover biological activity of VEGF-C. The X-ray structure of VEGF-C complexed with VEGFR-2 (or VEGFR-3)^16,60^ supports the notion that in a VEGF-C/VEGFR-3 complex, an MBP domain N-terminally to the VEGF homology domain would point away from the receptor and thus not interfere with receptor binding and activation. On the other hand, domains C-terminally to the VHD might point towards the cell surface and thus be more likely to interfere with receptor binding and/or activation, similar to the silk-homology domain, which keeps pro-VEGF-C inactive^5,32,61^. In the most straightforward scenario, these domains would be located between the extracellular domains 4 and 5 of VEGFR-3, which are instrumental in propagating the dimerization that is initiated by domains 1–3 towards the cell surface and interior^60^.

The total yield of VEGF-C remained modest (about 0.1 and 0.6 mg of active VEGF-C per liter of *E. coli* culture from Ni sepharose and amylose affinity chromatography, respectively). However, the yield was limited due to incomplete binding of VEGF-C to both resins due to partial or complete cleavage of the N-terminal hexahistidine or the MBP tag, which resulted in the flowthrough showing almost the same level of activity in the Ba/F3-VEGFR-3 assay as the input lysate (Figs. 6d, 7e). We could increase the yield by sixfold using amylose affinity chromatography. We estimate that alternative chromatographic methods such as using immobilized VEGFR-3^50^ could further increase the yield. Together with optimization of the production parameters, the use of *E. coli* could therefore be a viable replacement for the eukaryotic expression of VEGF-C. Even larger improvements could perhaps be achieved by deploying PDIs in the Origami strain using the MBP-VEGF-C fusion protein, a combination that was not tested in our study.

In addition to producing mature, active VEGF-C from a truncated cDNA, we also attempted to produce full-length VEGF-C (i.e., VEGF-C together with its N- and C-terminal propeptides). However, none of our attempts were successful. We speculate that the repetitive cysteine-rich Balbian Ring 3 protein (BR3) motif in the C-terminal domain of VEGF-C precludes the generation of any meaningful amounts of correctly folded protein. Even if correctly folded, such full-length protein would not be active unless concertedly cleaved by a preprotein convertase such as furin and a specific activating enzyme (ADAMTS3, KLK3, cathepsin D, plasmin, thrombin). Because both cleavages can happen in the supernatant of 293T cells^2^, we exposed the bacterial full-length VEGF-C to conditioned 293T cell supernatant, but could not detect any activation.

VEGF-C is the central molecule required for lymphatic growth. It has potential applications in vascular biology research as a supplement in lymphatic endothelial cell culture, and as a pharmacological agent in the treatment of lymphedema. VEGF-C has already been used—in the form of the adenoviral vector AdVEGF-C—in clinical studies to treat lymphedema^38^. While bacterial VEGF-C could be a cost-effective recombinant source of the growth factor for many in-vitro applications, glycosylated VEGF-C remains the first choice for in-vivo applications. Although mammalian-type N-glycosylation has been engineered into *E. coli*^62^, the routine delivery of glycosylated therapeutic proteins is currently achieved using viral vectors, mRNA drugs, or recombinant protein derived from a mammalian cell line.

## Materials and methods

### Clonings

#### CyDisCo

To produce the CyDisCo constructs, a synthetic, *E. coli*-codon-optimized cDNA coding for human VEGF-C (Phe32-Ser419 with mutation Cys137Ala) was used as a PCR template to generate the VEGF-C fragments listed in Table S1. The primers used for the amplification are listed in Table S2. VEGF-C genes were cloned into vector pJV84^28^ via digestion of parental vector and PCR product with NdeI/BamHI and ligation. All constructs have a C-terminal hexahistidine-tag after the BamHI restriction site, which translates into Gly-Ser.

#### Periplasmic expression

For periplasmic expression, we inserted the coding sequences for the pelB secretion signal (annealed primers 5′-GATCCGAATTAATTCCGATATCCATGGCCATCGCCGGCTGGGCAGCGAGGAGCAGCAGACCAGCAGCAGCGGTCGGCAGCAGGTATTTCA-3′ and 5′-TATGAAATACCTGCTGCCGACCGCTGCTGCTGGTCTGCTGCTCCTCGCTGCCCAGCCGGCGATGGCCATGGATATCGGAATTAATTCG-3′) into BamHI/NdeI-opened pET24b(+) (Novagen), resulting in pET24b(+)-pelB. All VEGF-C cDNAs were transferred from pET28b(+) to pET24b(+)-pelB as StyI fragments, except for the minor, mature form, which was amplified by PCR with primers 5′-CCGCGAAGACTTCATGACAGAAGAGACTATAAAATTTGC-3′ and 5′-CCGGATCCTCAACGTCTAATAATGGAATGAACTTG-3′ and transferred as NcoI/BamHI fragment (Table S1).

#### MBP-tagged expression

For MBP-tagged VEGF-C expression in the *E. coli* cytosol, all VEGF-C cDNAs were transferred from the periplasmic expression vector pET24(+)-pelB into a modified pMJ915 vector^63^. To create this modified expression vector (called pMJ915-MBP), the Cas9 CDS and the bom element of the pMJ915 vector had been deleted. All transfers happened via restriction enzyme cloning of PCR-amplified fragments (for cloning details see Table S1 and the supplemental plasmid maps).

#### Cytoplasmic expression

To produce the different forms of VEGF-C inclusion bodies in the *E. coli* cytosol for solubilization and folding, its cDNAs were cloned into pET28b(+) (Novagen; for cloning details see Table S1 and the supplemental plasmid maps).

All DNA constructs generated in this study were verified by Sanger sequencing.

### Cell lines

293T cells were obtained from ATCC (RRID: CVCL_0063). Stably transfected PAE cells expressing VEGFR-3^64^ and VEGFR-2^65^ and Ba/F3 cells stably expressing chimeric VEGFR-3^66^ and VEGFR-2^67^ were obtained directly from the reference laboratories. All the cells were grown in D-MEM containing 10% FCS.

### Antibodies

We used the following antibodies: anti-VEGF-C antiserum 6 (AS 6)^68^ and antiserum 882 (AS 882)^2^, Penta-His antibody (RRID:AB_2619735), anti-phosphotyrosine antibody PY20, anti-VEGFR-3 antibody 707^69^, anti-VEGFR-2 antibody RS-2^65^.

### Pulse chase

293T cells were transfected with the indicated constructs using JetPEI and grown for 36 h on 6-cm dishes to almost-confluency. Cells were starved for 30 min in met-/cys-deficient D-MEM 5% dialyzed FCS, after which 1 mL of labeling medium (met-/cys-deficient D-MEM supplemented with 20 µL EasyTag EXPRESS ^35^S/mL (Perkin Elmer) and 5% dialyzed FCS) was added to each dish for 2 h. Thereafter, cells were washed with warm PBS and 5 mL of chase medium was added (D-MEM 10% FCS + 1 mM cold L-methionine + 2 mM cold L-cysteine). At the indicated time points dishes were placed on ice, the medium was removed and the cells were washed with PBS and lysed. Analysis was performed by immunoprecipitating VEGF-C with anti-hVEGF-C antiserum^68^. The immunoprecipitated samples were resolved on a reducing SDS-PAGE and signals captured from dried gels with a phosphoimager plate and a Typhoon scanner.

### Glycosylation mutants

To produce the single and double glycosylation mutants of VEGF-C, N > Q substitution was done in the cDNA of VEGF-C at amino acid position N175 and/or N205. Equivalent N > W glycosylation mutants were also cloned. 293T cells were transfected with the mutant constructs in 6-well-plates using the calcium phosphate method followed by metabolic labeling. EasyTag EXPRESS ^35^S was used 8 µL/mL and cells were allowed to incorporate the label for 24 h. The supernatants and lysates were subjected to immunoprecipitation with VEGFR-2/Fc, VEGFR-3/Fc and AS882, respectively. The immunoprecipitated samples were resolved by 15% SDS-PAGE and the image of the dried gels was acquired with a Bas-1500 Bio Imaging Analyzer (Fujifilm).

### Protein production

#### CyDisCo

For CyDisCo expression, chemically competent MG1655 cells were transformed with pAGJ plasmid (AmpR) encoding respective VEGF-C fragments (Table S1) and with a second plasmid pMJS205 carrying CyDisCo components Erv1p and PDI. A single bacterial colony was picked from the transformation agar plate or from a restreaked frozen glycerol stock to inoculate 2 mL of LB containing 0.4% glucose and selection markers. Pre-culture was incubated for about 6 h at 37 °C at 250 rpm. 25 mL of freshly made chemically defined media was prepared in 250 mL shake flasks and inoculated with 250 µL of pre-culture. (One liter of chemically defined media was composed of 3.2 g of NH_4_Cl, 3.25 g glucose monohydrate, 11.1 g glycerol, 8.15 g lactose monohydrate, 1.2 g MgSO_4_.7H_2_O, 17.42 g of K_2_HPO_4_, 1.86 g of citric acid monohydrate, 100 mg of Fe III citrate, 0.23 mg of Na_2_MoO_4_·2H_2_O, 2.5 mg of CoCl_2_·6H_2_O, 15 mg of MnCl_2_·4H_2_O, 1.5 mg of CuCl_2_·2H_2_O, 3 mg of boric acid, 33.8 mg of zinc acetate.2H_2_O, and 12.5 mg of Na_2_-EDTA). The shaker flasks were sealed with air-permeable membrane AirOtop Enhanced Seals (Thomson) and the cultures were incubated o/n at 30 °C, 200 rpm (Infors HT incubator). The next day cultures were induced with 0.5 mM IPTG and incubated at 30 °C, 200 rpm for 24 h.

The bacteria were harvested by centrifugation at 5000*g* for 15 min at 4 °C. The supernatants were discarded, and the bacterial pellets were washed by resuspending in a total of 1/5 to 1/2 of the bacterial culture volume of cold bacterial harvest buffer (50 mM Tris/HCl, 2 mM EDTA, pH 8) followed by centrifugation at 10,000*g* for 10 min or 20,000 g for 1 min. The cells were then resuspended in 25 mL of lysis buffer (50 mM phosphate buffer, pH = 7.4 with 0.1 mg/mL lysozyme (lysozyme from chicken egg white, Sigma Aldrich) and 20 µg/mL DNase (DNase I from bovine pancreas, Roche)). The lysates were incubated at room temperature for 15 min and frozen at − 20 °C. The lysates were thawed after at least 16 h and total and soluble fractions + /− CyDisCo were resolved on a 12.5% SDS-PAGE gel.

### Expression for periplasmic targeting

For periplasmic expression, a 10-mL pre-culture was grown o/n at 37 °C in kanamycin- and tetracycline-supplemented LB medium (50 µg/mL and 12.5 µg/mL, respectively) of a single colony from the transformation agar plate. Subsequently, the o/n culture was diluted 1:100 into 25 mL LB medium in 250-mL Erlenmeyer flasks supplemented with MgSO_4_ to a final concentration of 0.5 mM. Bacteria were grown at 37 °C in an orbital shaker (250 rpm) until an OD_600_ of 0.5, and IPTG was added from a 100 mM stock to a final concentration of 100 µM. Incubation was continued for 3 h. Bacteria were harvested as described above for the CyDisCo expression.

The periplasmic extraction was performed based on cold osmotic shock with MgCl_2_^70^. Briefly, the bacterial pellet was washed with 85 µL PBS, centrifuged for 3 min at 20,000*g* and the supernatant was carefully removed. The cell pellet was then resuspended in 900 µL of cold spheroplast buffer (0.1 M Tris/HCl, 500 mM sucrose, 0.5 mM EDTA, pH 8). After 5 min incubation on ice and 3 min centrifugation at 20,000g at 4 °C, the supernatant was discarded. The bacterial pellet was then resuspended in 400 µL of cold 1 mM MgCl_2_ and after 15 s incubation on ice, 20 µL MgSO_4_ (20 mM) was added. Finally, centrifugation was performed as above and the periplasmic fraction (supernatant) was collected and stored at − 20 °C.

### Cytoplasmic expression

*E. coli* Origami (DE3) and BL21 (DE3) cells were transformed with the pET15b + or pMJ915 and pET28b + or pMJ915 expression plasmids, respectively. Single bacterial colonies were picked from the transformation agar plate and 5 mL o/n cultures were grown in LB medium with the corresponding antibiotic(s): 100 µg/mL ampicillin + 50 µg/mL kanamycin + 12.5 µg/mL tetracycline for Origami (DE3), and 50 µg/mL kanamycin for BL21 (DE3). The o/n cultures were diluted 1:100 into Terrific Broth (TB) medium. For analytical expression, 10-mL-cultures were grown in 50-mL Falcon tubes. Bacteria were grown at 37 °C in an orbital shaker (250 rpm) until an OD_600_ of 0.4–0.8, and IPTG was added from a 100 mM stock to a final concentration of 0.5 mM. Incubation was continued for 20 h at 25 °C. Bacteria were harvested as described above for the CyDisCo expression.

Following protein production, the frozen *E. coli* were thawed on ice and resuspended in 1 mL PBS. After mixing 100 µL of the cell suspension with 100 µL of 2×Laemmli sample buffer, cells were sonicated in an ice-water bath (Fisher Scientific FB120, 1/8 in. tip, 8 cycles of 6 s/10 s burst/cooling at 50% amplitude). The bacterial lysate was centrifuged at 13,000*g* for 30 min at 4 °C and the supernatant and cell pellets were collected and stored at − 20 °C.

### Cytoplasmic expression for refolding

A single bacterial colony was picked from the transformation agar plate or a restreaked frozen glycerol stock and a 5 mL o/n culture was grown in LB medium containing 50 µg/mL kanamycin. The o/n culture was diluted 1:100–1:1000 into Terrific Broth (TB) medium. For analytical batches, 10-mL-cultures were grown in 50-mL Falcon tubes, and for production batches, 250-mL-cultures were grown in 1 L Erlenmeyer flasks. Bacteria were grown at 37 °C in an orbital shaker (250 rpm) until an OD_600_ of 0.4–0.8, and IPTG was added from a 100 mM stock to a final concentration of 1 mM. Incubation was continued for 3.5 to 4 h. Bacteria were harvested as described above for the CyDisCo expression and stored at − 80 °C.

### Bacterial lysis for refolding

Following protein production, the thawed *E. coli* pellet was washed by resuspending it in bacterial harvest buffer (25 mL for each gram of bacterial pellet, but maximally 50 mL) and centrifuging at 8000*g* for 15 min at 4 °C. After this, they were lysed in bacterial lysis buffer (50 mM Tris/HCl pH 8.0, 2 mM EDTA, 10 mM ß-mercaptoethanol, 5% glycerol; 5 mL for each gram of bacterial pellet), which was complemented with lysozyme (1 mg lysozyme to each gram of bacterial pellet from a 10 mg/mL lysozyme solution in bacterial harvest buffer), and the mixture was stirred at RT for 20 min.

After this, 1/9 volume of the detergent mix (10% NP-40 and 0.5% NaDOC) was added to the mixture (565 µL for each gram bacterial pellet), and the stirring was continued for another 20 min. Then 250 units of DNaseI were added for each gram bacterial pellet and stirring continued for 30 min at 30 °C. Cells were then sonicated in an ice-water bath (Fisher Scientific FB120, 1/8 in. tip, 3–6 cycles of 10 s/20 s burst/cooling at 100% amplitude). The maximal volume for sonication was 8 mL; larger volumes were split into aliquots and sonicated separately.

### Inclusion body preparation

The bacterial lysate was centrifuged at 13,000*g* for 30 min at 4 °C. The pellet was resuspended in bacterial lysis buffer. The lysozyme, DNaseI, and detergent treatments and centrifugation were repeated up to 4 times. To remove lysozyme, DNaseI, and detergent, the inclusion body pellet was three times resuspended in bacterial lysis buffer and centrifuged at 13,000*g* (except for the last centrifugation, which was performed at 10,000*g*). For storage, inclusion body pellets were frozen at − 70 °C before the last centrifugation step.

### Solubilization of inclusion bodies

Each gram of isolated inclusion bodies was resuspended in 4 mL of solubilization buffer (6M Gu-HCl, 20 mM Tris/HCl pH 8.0, 5 mM EDTA, 10 mM DTT) and incubated for 2 h at RT under agitation. To remove unsolubilized material, the reaction was centrifuged using an SW41Tl rotor at 20,000 rpm (~ 46,000*g*) for 30 min and the supernatant was transferred to a new tube.

### Protein folding

In the first screen, we tested 32 different conditions for their ability to support the folding of a minimal form of VEGF-C, which consisted only of the essential VHD. Half of these conditions were identical to the ones reported by^71^, the rest were identical to reportedly working folding conditions for other proteins from the same (PDGF/VEGF) protein family (see Table S3a). Based on the two most efficient folding conditions, we set up two additional screens to optimize the conditions (Tables S3b and S3c). A fourth folding screen was then performed using selected conditions from the previous screens (see Table S3d), but this time we used both the minimal version of VEGF-C as well as the two most abundant naturally occurring forms of mature VEGF-C: the major, mature form (produced by furin- and ADAMTS3-cleavage) and the minor, mature form (produced by furin- and plasmin-cleavage) (for a review about the different VEGF-C cleavages, see^72^). Refolding was performed by adding 50 µL of solubilized inclusion bodies to 1 mL refolding buffer and incubating the reaction at + 4 °C under gentle agitation o/n. After refolding, samples were dialyzed against three buffer changes of 100 volumes of PBS-T (0.05% Tween 20) and a final buffer change against PBS. Protein that had precipitated during dialysis was removed by centrifugation at 20,800*g* for 30 min.

### Assays for biologically active, correctly folded VEGF-C

For the first three folding screens, we assayed the successful refolding by testing the ability of the samples to induce phosphorylation of the main VEGF-C receptor (VEGFR-3) as described previously^46^. In a nutshell, serum-starved PAE cells stably transfected with VEGFR-3 were exposed to a 1:10 dilution of the refolded samples for 5 min, after which the cells were placed on ice, lysed, and analyzed for phosphorylated VEGFR-3 after polyacrylamide gel electrophoresis and Western blotting (data not shown). The refolded samples were used at a concentration of 10%.

The samples of the fourth folding screen were assayed using a chimeric receptor consisting of the extracellular domain of human VEGFR-3 fused to the transmembrane and intracellular domain of the erythropoietin receptor (hVEGFR-3/EpoR) as described previously^66^. Since we had never found any discrepancies between the phosphorylation assays using native VEGFR-3 and the Ba/F3-hVEGFR-3/EpoR bioassays when comparing dimeric VEGF growth factors^6,46^, we used the chimeric bioassay for the rest of these studies, notably for assaying the VEGF-C targeted to the periplasm and the MBP-tagged VEGF-C.

### Purification of the refolded VEGF-C

The dialyzed and cleared refolded material was concentrated on a YM-3 Centricon device according to the instructions of the manufacturer (Millipore) and resolved on a Superdex 75 10/300 GL size exclusion chromatography column using PBS as running buffer and a flow rate between 0.5 and 0.6 mL/min. Calibration of the column was performed with a blend of BSA (New England Biolabs, #B7000), carbonic anhydrase (Sigma C7025) and RNaseA (Qiagen #19101) in PBS (each protein at 1.25 mg/mL). The dimeric (11.5–13.5 mL) as well as the monomeric (13.5–15.5 mL) fractions showed both biological activity in the Ba/F3-VEGFR-3/EpoR assay.

### Purification of the MBP/VEGF-C fusion protein

MBP-tagged minimal mature VEGF-C in the modified pMJ915 vector was transformed into *E. coli* Origami (DE3) cells and a single bacterial colony was picked from the transformation agar plate and a 10 mL o/n culture was grown in LB medium containing 100 µg/mL ampicillin, 50 µg/mL kanamycin, and 12.5 µg/mL tetracycline. The o/n culture was diluted 1:100 into 1 L of LB medium. Bacteria were grown at 37 °C in an orbital shaker (250 rpm) until an OD_600_ of 0.4–0.8 and induced with 0.5 mM IPTG. Incubation was continued for 20 h at 25 °C. Bacteria were harvested as described above for the CyDisCo expression.

Following protein production, the thawed bacterial pellet was resuspended in lysis buffer (50 mM NaH_2_PO_4_, 300 mM NaCl) complemented with 0.1 mg/mL lysozyme and protease inhibitor (Pierce™ Protease Inhibitor Tablets, EDTA-free), and kept on ice for 30 min. Cells were sonicated in an ice-water bath (Fisher Scientific FB120, ¼ inch tip, 8–10 cycles of 10 s/20 s burst/cooling at 100% amplitude). The bacterial lysate was centrifuged at 13,000*g* for 30 min at 4 °C and the supernatant was collected for purification.

For the purification of his-tagged proteins, 1 mL of Ni sepharose (Excel, Cytiva) was added to the lysate for o/n batch binding at 4 °C. The Ni sepharose was loaded to a column, washed with 10 mM imidazole, and eluted with 500 mM imidazole. For amylose affinity purification, 1 mL of amylose resin (NEB) was added to the lysate for o/n batch binding. The amylose resin was then washed 5 times with binding buffer and eluted with 10 mM maltose. The peak fraction or concentrated peak fractions (500 µL) were further resolved on a Superdex 200 10/300 GL size exclusion chromatography column using PBS as a running buffer and a flow rate between 0.4 and 0.5 mL/min. The concentrated fractions after elution with maltose were treated with 5 µL TEV protease (2.4 mg/mL; purified in-house) to remove the MBP tag before SEC. The fractions were analyzed by SDS-PAGE followed by Coomassie/silver staining and Western blotting. The biological activity of the resolved fractions was analyzed using Ba/F3-VEGFR-3/EpoR assay.

### Activation of bacterial full-length VEGF-C using 293T cells

293T cells on a 12-well-plate were 70% confluent when the medium was changed to 400 µL *E. coli* cell lysates that were filtered through 0.45 µm syringe filter units. The lysates were obtained by sonication of Origami (DE3) resuspended in serum-free D-MEM. 24 h later the conditioned media were collected and supernatants were used in Ba/F3 assay at a maximal concentration of 20% of final culture volume.

### Statistical analysis

Data are presented as mean ± SD. Data were analyzed using GraphPad Prism statistical analysis software (version 8.4.2 for Windows, GraphPad Software, San Diego, California USA, www.graphpad.com), OriginPro for statistical analysis (version 9.7), and Numbers (version 10.3.9). The number of replicates is mentioned in the respective figure legends.

## Supplementary Information

Supplementary Information 1.

Supplementary Information 2.

## Data Availability

All data generated or analyzed during this study are included in this published article and its supplementary figures and tables.

## References

[1] Rauniyar K, Jha SK, Jeltsch M (2018). Biology of vascular endothelial growth factor C in the morphogenesis of lymphatic vessels. Front. Bioeng. Biotechnol..

[2] Joukov V (1997). Proteolytic processing regulates receptor specificity and activity of VEGF-C. EMBO J..

[3] Siegfried G (2003). The secretory proprotein convertases furin, PC5, and PC7 activate VEGF-C to induce tumorigenesis. J. Clin. Invest..

[4] McColl BK (2003). Plasmin activates the lymphangiogenic growth factors VEGF-C and VEGF-D. J. Exp. Med..

[5] Jeltsch M (2014). CCBE1 enhances lymphangiogenesis via a disintegrin and metalloprotease with thrombospondin motifs-3–mediated vascular endothelial growth factor-C activation. Circulation.

[6] Jha SK (2019). KLK3/PSA and cathepsin D activate VEGF-C and VEGF-D. eLife.

[7] Lim L (2019). Hemostasis stimulates lymphangiogenesis through release and activation of VEGFC. Blood.

[8] Kase S (2010). αB-crystallin regulation of angiogenesis by modulation of VEGF. Blood.

[9] Ozawa K (2001). Expression of the oxygen-regulated protein ORP150 accelerates wound healing by modulating intracellular VEGF transport. J. Clin. Invest..

[10] Christinger HW (1996). Crystallization of the receptor binding domain of vascular endothelial growth factor. Proteins Struct. Funct. Bioinform..

[11] Christinger HW, Fuh G, de Vos AM, Wiesmann C (2004). The crystal structure of placental growth factor in complex with domain 2 of vascular endothelial growth factor receptor-1. J. Biol. Chem..

[12] Iyer S (2001). The crystal structure of human placenta growth factor-1 (PlGF-1), an angiogenic protein, at 2.0 Å resolution. J. Biol. Chem..

[13] Iyer S, Scotney PD, Nash AD, Ravi Acharya K (2006). Crystal structure of human vascular endothelial growth factor-B: Identification of amino acids important for receptor binding. J. Mol. Biol..

[14] Muller YA (1997). Vascular endothelial growth factor: crystal structure and functional mapping of the kinase domain receptor binding site. Proc. Natl. Acad. Sci. USA.

[15] Scrofani SD, Fabri LJ, Xu P, Maccarone P, Nash AD (2000). Purification and refolding of vascular endothelial growth factor-B. Protein Sci. Publ. Protein Soc..

[16] Leppänen V-M (2010). Structural determinants of growth factor binding and specificity by VEGF receptor 2. Proc. Natl. Acad. Sci..

[17] Leppänen V-M (2011). Structural determinants of vascular endothelial growth factor-D receptor binding and specificity. Blood.

[18] Bessette PH, Åslund F, Beckwith J, Georgiou G (1999). Efficient folding of proteins with multiple disulfide bonds in the *Escherichia coli* cytoplasm. Proc. Natl. Acad. Sci..

[19] Hatahet F, Nguyen VD, Salo KE, Ruddock LW (2010). Disruption of reducing pathways is not essential for efficient disulfide bond formation in the cytoplasm of *E. coli*. Microb. Cell Factories.

[20] Nguyen VD (2011). Pre-expression of a sulfhydryl oxidase significantly increases the yields of eukaryotic disulfide bond containing proteins expressed in the cytoplasm of *E. coli*. Microb. Cell Factories.

[21] de Marco A (2009). Strategies for successful recombinant expression of disulfide bond-dependent proteins in *Escherichia coli*. Microb. Cell Factories.

[22] Berkmen M (2012). Production of disulfide-bonded proteins in *Escherichia coli*. Protein Expr. Purif..

[23] Ruano-Gallego D, Fraile S, Gutierrez C, Fernández LÁ (2019). Screening and purification of nanobodies from *E. coli* culture supernatants using the hemolysin secretion system. Microb. Cell Factories.

[24] Matos CFRO (2014). Efficient export of prefolded, disulfide-bonded recombinant proteins to the periplasm by the Tat pathway in *Escherichia coli* CyDisCo strains. Biotechnol. Prog..

[25] Rasmussen JR (1992). Effect of glycosylation on protein function. Curr. Opin. Struct. Biol..

[26] Karpanen T (2006). Functional interaction of VEGF-C and VEGF-D with neuropilin receptors. FASEB J..

[27] Derman AI, Prinz WA, Belin D, Beckwith J (1993). Mutations that allow disulfide bond formation in the cytoplasm of *Escherichia coli*. Science.

[28] Gaciarz A (2016). Systematic screening of soluble expression of antibody fragments in the cytoplasm of *E. coli*. Microb. Cell Factories.

[29] Dagar VK, Adivitiya, Khasa YP (2017). High-level expression and efficient refolding of therapeutically important recombinant human Interleukin-3 (hIL-3) in *E. coli*. Protein Expr. Purif..

[30] Lundell D (1990). Cytoplasmic and periplasmic expression of a highly basic protein, human interleukin 4, in *Escherichia coli*. J. Ind. Microbiol..

[31] Lebendiker M, Danieli T (2017). Purification of proteins fused to maltose-binding protein. Methods Mol Biol..

[32] Joukov V (1997). Proteolytic processing regulates receptor specificity and activity of VEGF-C. EMBO J..

[33] Riggs AD (2021). Making, cloning and expression of human insulin genes in bacteria: The path to Humulin. Endocr. Rev..

[34] Jayson GC, Kerbel R, Ellis LM, Harris AL (2016). Antiangiogenic therapy in oncology: current status and future directions. Lancet.

[35] Toi M (2002). Significance of vascular endothelial growth factor (VEGF)/soluble VEGF receptor-1 relationship in breast cancer. Int. J. Cancer.

[36] Demirovic A (2005). Targeting human cancer cells with VEGF receptor-2-directed liposomes. Oncol. Rep..

[37] Bainbridge JWB (2003). A peptide encoded by exon 6 of VEGF (EG3306) inhibits VEGF-induced angiogenesis in vitro and ischaemic retinal neovascularisation in vivo. Biochem. Biophys. Res. Commun..

[38] Hartiala P (2020). Phase 1 LymfactinⓇ Study: short-term safety of combined adenoviral VEGF-C and lymph node transfer treatment for upper extremity lymphedema. J. Plast. Reconstr. Aesthet. Surg..

[39] Mazor Y, Van Blarcom T, Mabry R, Iverson BL, Georgiou G (2007). Isolation of engineered, full-length antibodies from libraries expressed in *Escherichia coli*. Nat. Biotechnol..

[40] Pizarro SA (2010). High-yield expression of human vascular endothelial growth factor VEGF165 in *Escherichia coli* and purification for therapeutic applications. Protein Expr. Purif..

[41] Siemeister G (1996). Expression of biologically active isoforms of the tumor angiogenesis factor VEGF in *Escherichia coli*. Biochem. Biophys. Res. Commun..

[42] Kim YS (2021). Effective production of human growth factors in *Escherichia coli* by fusing with small protein 6HFh8. Microb. Cell Factories.

[43] Seyedarabi A, Cheng L, Zachary I, Djordjevic S (2013). Production of soluble human vascular endothelial growth factor VEGF-A165-heparin binding domain in *Escherichia coli*. PLoS ONE.

[44] Oh S-J (1997). VEGF and VEGF-C: Specific induction of angiogenesis and lymphangiogenesis in the differentiated avian chorioallantoic membrane. Dev. Biol..

[45] Achen MG (1998). Vascular endothelial growth factor D (VEGF-D) is a ligand for the tyrosine kinases VEGF receptor 2 (Flk1) and VEGF receptor 3 (Flt4). Proc. Natl. Acad. Sci..

[46] Jeltsch M (2006). Vascular endothelial growth factor (VEGF)/VEGF-C mosaic molecules reveal specificity determinants and feature novel receptor binding patterns. J. Biol. Chem..

[47] Davydova N (2012). Preparation of human vascular endothelial growth factor-D for structural and preclinical therapeutic studies. Protein Expr. Purif..

[48] BioVision. *Datasheet: Recombinant Human VEGF-C* (2010).

[49] Chiu J, Wong JWH, Gerometta M, Hogg PJ (2014). Mechanism of dimerization of a recombinant mature vascular endothelial growth factor C. Biochemistry.

[50] Joukov V (1996). A novel vascular endothelial growth factor, VEGF-C, is a ligand for the Flt4 (VEGFR-3) and KDR (VEGFR-2) receptor tyrosine kinases. EMBO J..

[51] von Einem S, Schwarz E, Rudolph R (2010). A novel TWO-STEP renaturation procedure for efficient production of recombinant BMP-2. Protein Expr. Purif..

[52] Reigstad LJ (2003). Platelet-derived growth factor (PDGF)-C, a PDGF family member with a vascular endothelial growth factor-like structure. J. Biol. Chem..

[53] Tuan T-L (1996). Engineering, expression and renaturation of targeted TGF-beta fusion proteins. Connect. Tissue Res..

[54] Obukowicz MG (1990). Secretion of active kringle-2-serine protease in *Escherichia coli*. Biochemistry.

[55] Leppanen V-M (2011). Structural determinants of vascular endothelial growth factor-D receptor binding and specificity. Blood.

[56] Uusitalo, E. *Improvement of the Quality of Insect-Cell-Derived, Recombinant Pro-VEGF-C* (2015).

[57] Kaplan O (2016). Enhanced mitogenic activity of recombinant human vascular endothelial growth factor VEGF121 expressed in *E. coli* Origami B (DE3) with molecular chaperones. PLOS ONE.

[58] Go Y-M, Jones DP (2008). Redox compartmentalization in eukaryotic cells. Biochim. Biophys. Acta.

[59] Mohanraj D, Olson T, Ramakrishnan S (1995). A novel method to purify recombinant vascular endothelial growth factor (VEGF121) expressed in yeast. Biochem. Biophys. Res. Commun..

[60] Leppänen V-M (2013). Structural and mechanistic insights into VEGF receptor 3 ligand binding and activation. Proc. Natl. Acad. Sci..

[61] Jha SK (2017). Efficient activation of the lymphangiogenic growth factor VEGF-C requires the C-terminal domain of VEGF-C and the N-terminal domain of CCBE1. Sci. Rep..

[62] Valderrama-Rincon JD (2012). An engineered eukaryotic protein glycosylation pathway in *Escherichia coli*. Nat. Chem. Biol..

[63] Lin S, Staahl BT, Alla RK, Doudna JA (2014). Enhanced homology-directed human genome engineering by controlled timing of CRISPR/Cas9 delivery. eLife.

[64] Pajusola K (1994). Signalling properties of FLT4, a proteolytically processed receptor tyrosine kinase related to two VEGF receptors. Oncogene.

[65] Waltenberger J, Claesson-Welsh L, Siegbahn A, Shibuya M, Heldin CH (1994). Different signal transduction properties of KDR and Flt1, two receptors for vascular endothelial growth factor. J. Biol. Chem..

[66] Achen MG (2000). Monoclonal antibodies to vascular endothelial growth factor-D block its interactions with both VEGF receptor-2 and VEGF receptor-3. Eur. J. Biochem..

[67] Stacker SA (1999). A mutant form of vascular endothelial growth factor (VEGF) that lacks VEGF receptor-2 activation retains the ability to induce vascular permeability. J. Biol. Chem..

[68] Baluk P (2005). Pathogenesis of persistent lymphatic vessel hyperplasia in chronic airway inflammation. J. Clin. Invest..

[69] Pajusola K, Aprelikova O, Armstrong E, Morris S, Alitalo K (1993). Two human FLT4 receptor tyrosine kinase isoforms with distinct carboxy terminal tails are produced by alternative processing of primary transcripts. Oncogene.

[70] Malherbe G, Humphreys DP, Davé E (2019). A robust fractionation method for protein subcellular localization studies in *Escherichia coli*. Biotechniques.

[71] Chen G-Q, Gouaux E (1997). Overexpression of a glutamate receptor (GluR2) ligand binding domain in *Escherichia coli*: Application of a novel protein folding screen. Proc. Natl. Acad. Sci. USA.

[72] Lackner M, Schmotz C, Jeltsch M (2019). The proteolytic activation of vascular endothelial growth factor-C. Lymphol. Forsch. Prax..

