# Supplementary material for: Bioactive VEGF-C from E. coli
Source: Sci Rep. 2022 Oct 28;12:18157. doi: 10.1038/s41598-022-22960-0 (PMC9616921; doi:10.1038/s41598-022-22960-0)
Supplement: Supplementary file 1 — Supplementary Information 1. [file 41598_2022_22960_MOESM1_ESM.pdf]

Bioactive VEGF-C from *E. coli*

Khushbu Rauniyar*[1], Soheila Akhondzadeh*[1], Anna Gąciarz[2], Jaana Künnapuu[1], Michael Jeltsch[1,2,3]

[1]Drug Research Program, University of Helsinki, Finland; [2]Individualized Drug Therapy Research Program, University of Helsinki, Finland; [3]Wihuri Research Institute, Helsinki, Finland

Supplementary Materials

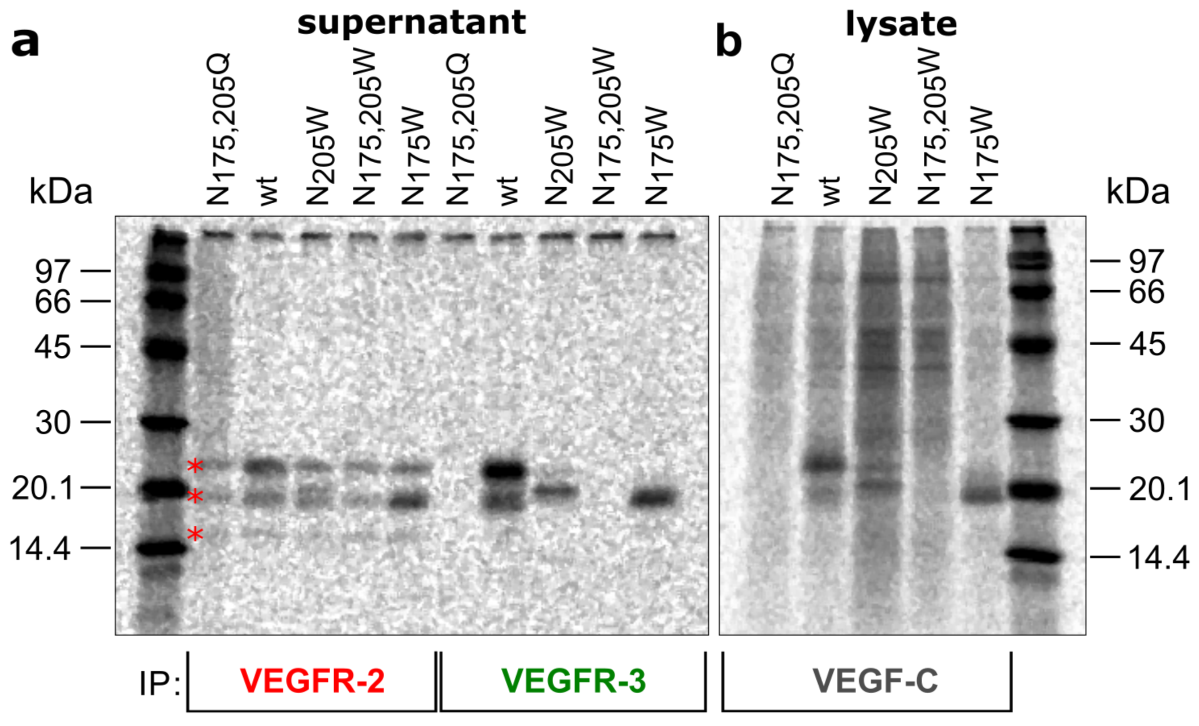

Supplementary Figure 1. Single glycosylation mutants (N>W) of mature VEGF-C expressed in 293T cells can bind VEGFR-2 and VEGFR-3, whereas double glycosylation

mutants have no expression.

Red asterisks denote 3 distinct bands of the isoforms of VEGF-A endogenously expressed by 293T cells.

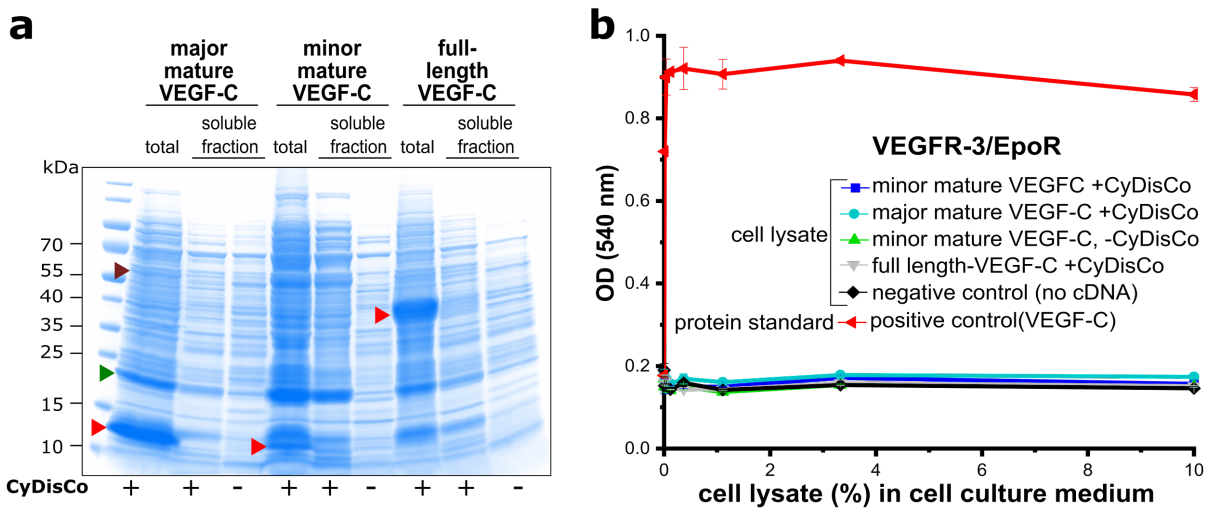

Supplementary Figure 2. Mature forms and full-length VEGF-C co-expressed with CyDisCo system in the cytoplasm of *E. coli* strain MG1655 aggregated into inclusion bodies.

The respective VEGF-C bands are indicated by the red arrows. The CyDisCo components, Erv1p (22kDa) and PDI (55kDa), are indicated by the green and brown arrows, respectively. Interestingly, PDI expression level varies significantly depending on the VEGF-C variant expressed.

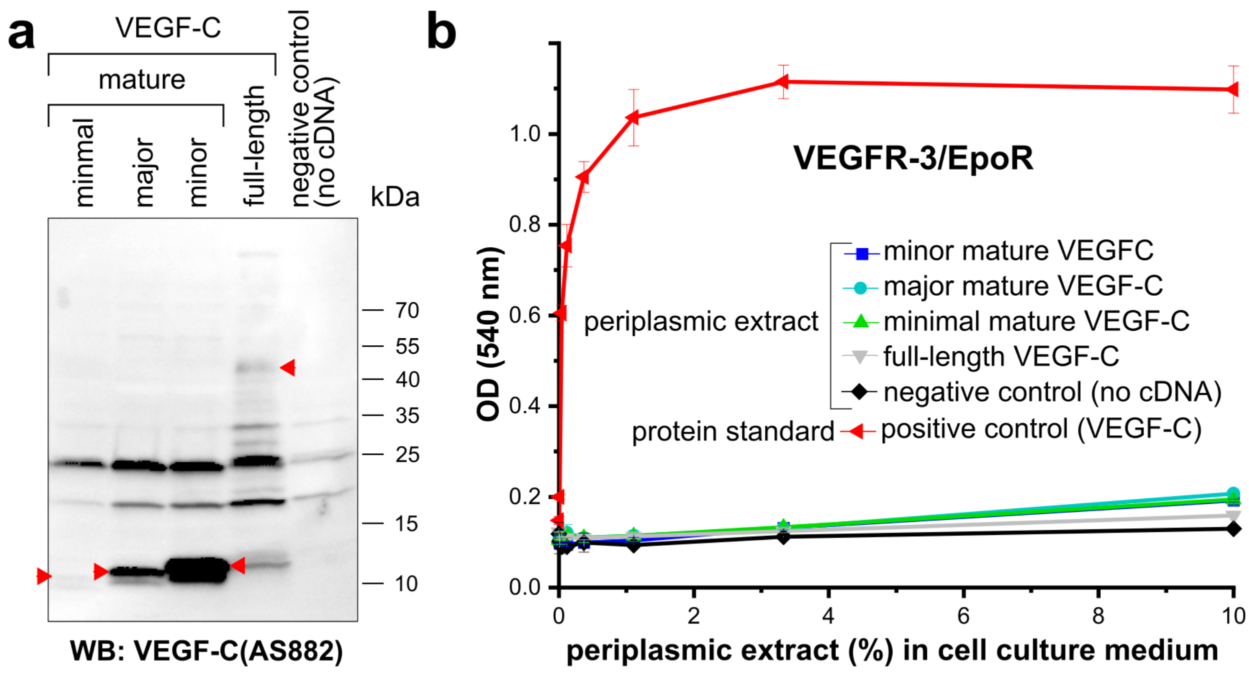

Supplementary Figure 3. Expression and activity of periplasmic VEGF-C. (a) Mature forms and full-length VEGF-C expressed in the periplasm of *E. coli* BL21 (DE3) strain (b) did not show significant activity in the Ba/F3-VEGFR-3/EpoR assay. However, the amounts of the minimal and the full-length form of VEGF-C retrieved from the periplasm remained well below those of the two naturally occurring mature forms of VEGF-C (major and minor mature VEGF-C). This could result from an increased flux in the system and hence expression from a weaker promoter such as tac might be beneficial. (n=2) Error bars indicate ±SD. The expected sizes for the minimal, major, and minor mature VEGF-C and full-length VEGF-C are ~11.7 kDa, ~13.2 kDa, ~14.2 kDa, and ~43.8 kDa, respectively. Red arrows indicate the respective VEGF-C bands.

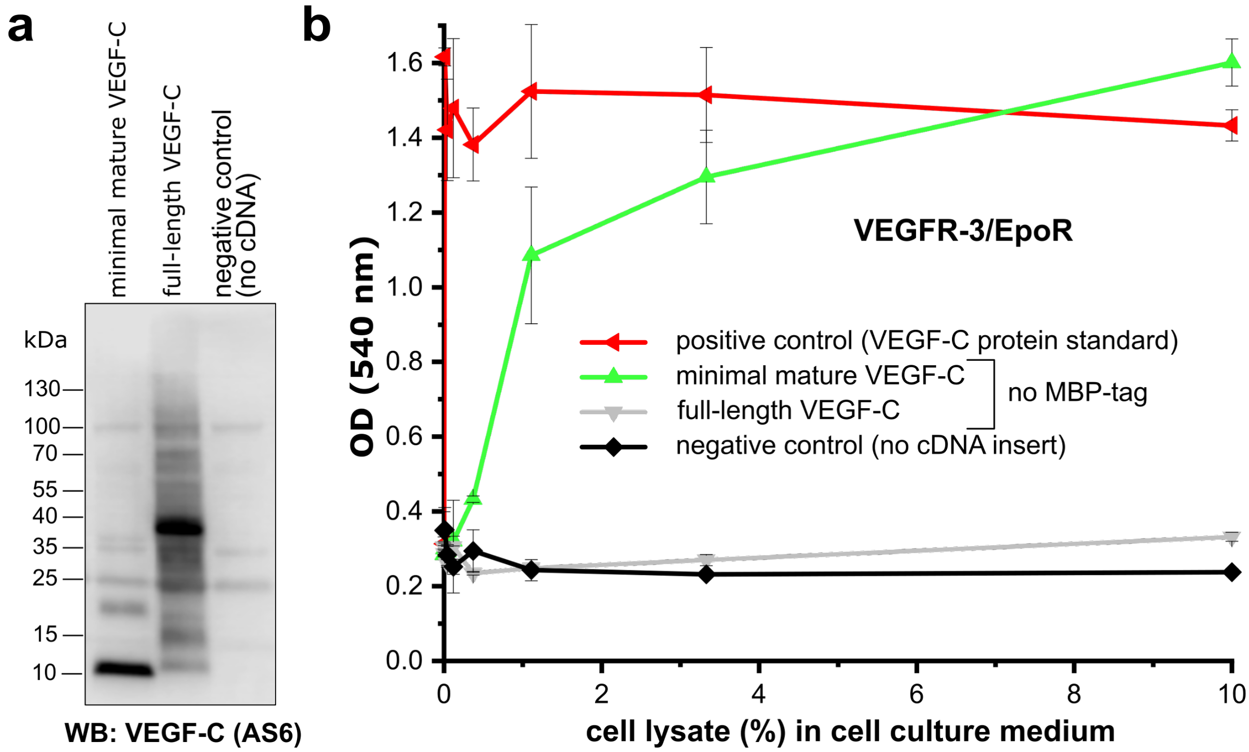

Supplementary Figure 4. Expression and activity of untagged minimal mature and full-length VEGF-C. When expressed in the cytoplasm of *E. coli* Origami (DE3), untagged minimal mature VEGF-C does show some biological activity. However, a semi-quantitative comparison with the MBP-tagged version of the same protein shows that the MBP-tagging results in significantly enhanced biological activity (Figure 5). (n=2) Error bars indicate ±SD.

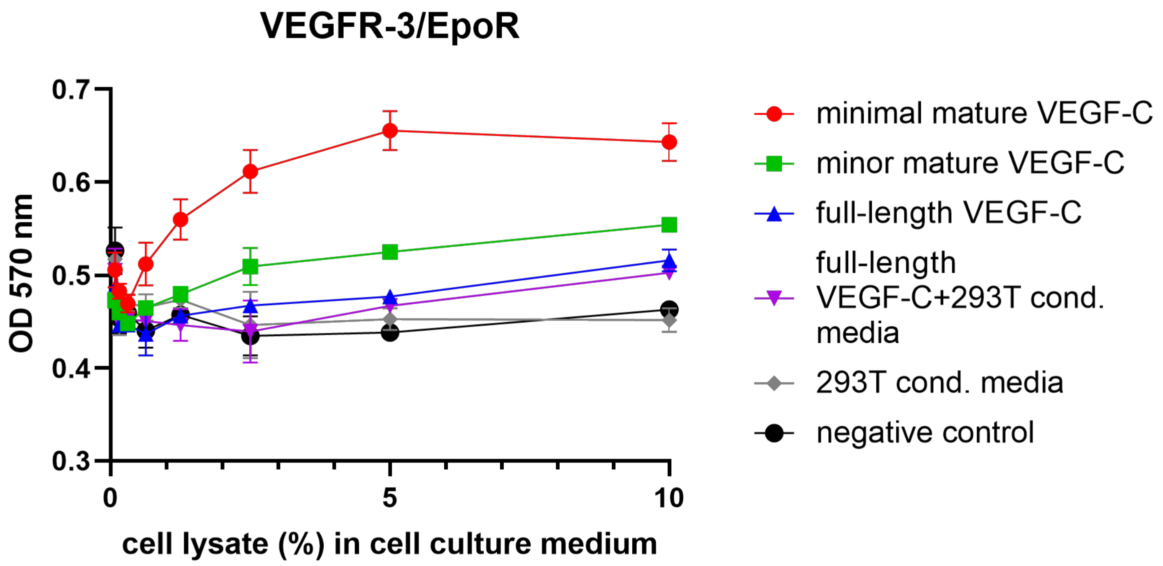

Supplementary Figure 5. MBP-tagged full-length VEGF-C expressed in *E. coli* Origami (DE3) does not get activated by exposure to 293T conditioned media.

While the minimal and minor mature forms of VEGF-C show activity in the Ba/F3 VEGFR-3/EpoR bioassay, full-length VEGF-C treated with conditioned 293T media does not perform better in the assay than untreated full-length VEGF-C. The line graph was generated using GraphPad Prism version 8.2.4 for Windows, GraphPad Software, San Diego, California USA, www.graphpad.com (n=2) Error bars indicate ±SD.

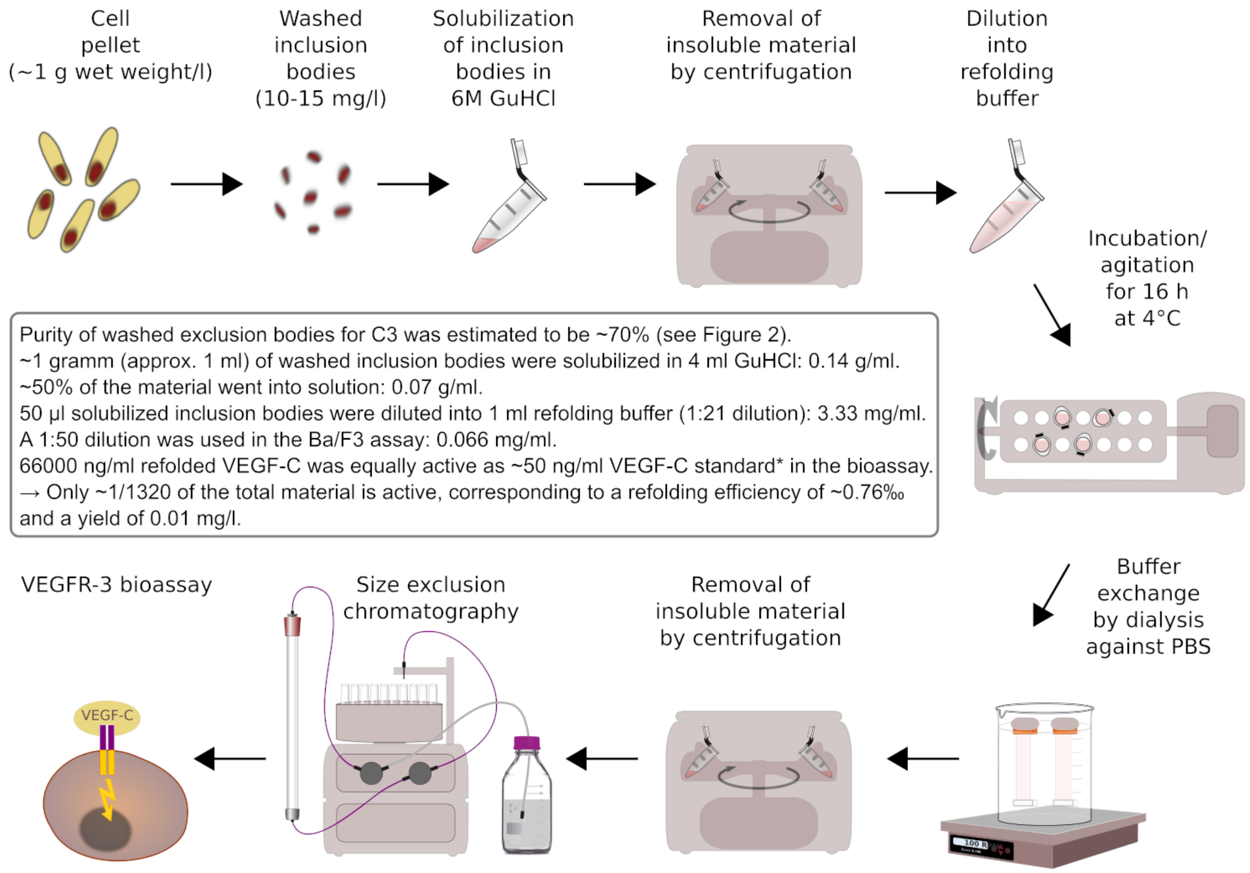

Supplementary Figure 6. Flow chart of VEGF-C refolding and calculation of refolding efficiency. The comparison with the VEGF-C standard (25) was done assuming near 100% activity of the VEGF-C standard (1.6 ng/ml < EC_{50} < 4 ng/ml).

Supplemental Table 1. Constructs and bacterial strains used in this study

S.N.	Construct	Details	Theoretical MW (kDa)	Selection	References	Lab-internal DNA prep number	Lab-internal DNA glycerol stock number
**I.**	**Constructs**						
**A.**	**Constructs for cytosolic expression in BL21 (DE3)**						
1.	pET28b(+)	empty (does not produce anything)	-	KanR	This study	247	1472
2.	pET28b(+)-minor_mature_VEGF-C	minor mature form of human VEGF-C, MT103-R227*	14.2	KanR	This study	795	1815
3.	pET28b(+)-major_mature_VEGF-C	major mature form of human VEGF-C, MA112-R227*	13.2	KanR	This study	796	1814
4.	pET28b(+)-minimal_mature_VEGF-C	minimal mature form of human VEGF-C (corresponds to VEGF-A109), MA112-D216*	11.8	KanR	This study	777	1797
5.	pET28b(+)-full length VEGF-C	full VEGF-C cDNA excl. signal peptide, untagged, ME33-S419*	43.8	KanR	This study	2515	4050
**B.**	**Constructs for cytosolic expression in Origami (DE3)**						
1.	pET15b(+)-minimal_mature_VEGF-C	minimal mature form of VEGF-C, MA112-D216*	11.8	AmpR	This study	285	514
**C.**	**Constructs for MBP-tagged VEGF-C production**						
1.	pMJ915-MBP-minor_mature_VEGF-C	minor mature form of human VEGF-C incl. solubility tag MBP , MT103-R227*		AmpR	This study	2552	4128
2.	pMJ915-MBP-major_mature_VEGF-C	major mature form of human VEGF-C incl. solubility tag MBP, MA112-R227*		AmpR	This study	2553	4129
3.	pMJ915-MBP-minimal_mature_VEGF-C	minimal mature form of human VEGF-C incl. solubility tag MBP (corresponds to VEGF-A109), MA112-D216*		AmpR	This study	2554	4130
4.	pMJ915-MBP-full length VEGF-C	full VEGF-C cDNA excl. signal peptide, including solubility tag MBP, ME33-S419*		AmpR	This study	2555	4131
**D.**	**Constructs for CyDisCo expression**						
1.	pAGJ-major_mature_VEGF-C	Mature chain, A112-R227 (C137A)-GSH6	14.2	AmpR	This study	2468	3919
2.	pAGJ-minimal_mature_VEGF-C	Crystallizable fragment, S101-L215	13.9	AmpR	This study	2474	3958
3.	pAGJ-full length VEGF-C	AA-Full length, A30-S419 (C137A)-GSH6	45.1	AmpR	This study	2473	3956
4.	pMJS205	expresses coErv1p from Saccharomycces cerevisiae and human coPDI	PDI: 55; Erv1p: 21.6	ChlR	Hatahet F. et. al, 2010	2450	3857
**E.**	**Constructs for periplasmic expression**						
1.	pET24(+)-minor mature VEGF-C	Minor mature form of human VEGF-C, MT103-R227*, incl pelB tag	14.2	KanR	This study	2530	4096
2.	pET24 (+)-majoe_mature_VEGF-C	Major mature form of human VEGF-C, MA112-R227*, incl pelB tag	13.2	KanR	This study	2537	4103
3.	pET24 (+)-minimal_mature_VEGF-C	Minimal mature form of human VEGF-C (corresponds to VEGF-A109), MA112-D216*, incl pelB tag	11.7	KanR	This study	2534	4100
4.	pET24 (+) full length VEGF-C	Full human VEGF-C cDNA excl. signal peptide, incl pelB tag, ME33-S419*	43.8	KanR	This study	2532	4098
**F.**	**Constructs for glycosylation mutants**						
1.	pSecTagI-IgKSP-dNdC-hVEGF-C-N175W-H6	mature form of VEGF-C (T103-L215) with single N175W mutation	16.2	AmpR	This study	468	1148
2.	pSecTagI-IgKSP-dNdC-hVEGF-C-N205W-H6	mature form of VEGF-C (T103-L215) with single N205W mutation	16.2	AmpR	This study	466	1144
3.	pSecTagI-IgKSP-dNdC-hVEGF-C-N175, 205W-H6	mature form of VEGF-C (T103-L215) with double N175, 205W mutations	16.2	AmpR	This study	467	1146
4.	pSecTagI-IgKSP-dNdC-hVEGF-C-N175Q-H6	mature form of VEGF-C (T103-L215) with single N175Q mutation	16.2	AmpR	This study	395	872
5.	pSecTagI-IgKSP-dNdC-hVEGF-C-N205Q-H6	mature form of VEGF-C (T103-L215) with single N205Q mutation	16.2	AmpR	This study	446	1175
6.	pSecTagI-IgKSP-dNdC-hVEGF-C-N175, 205Q-H6	mature form of VEGF-C (T103-L215) with double N175, 205Q mutations	16.2	AmpR	This study	445	1174
7.	pSecTagI-IgKSP-dNdC-hVEGF-C-H6	mature form of wild-type VEGF-C (T103-L215)	16.2	AmpR	This study	406	942
**II.**	**Bacterial strains**						
1.	Top 10	General cloning host; Genotype: F- mcrA Δ(mrr-hsdRMS-mcrBC) φ80lacZΔM15 ΔlacX74 nupG recA1 araD139 Δ(ara-leu)7697 galE15 galK16 rpsL(StrR) endA1 λ-		-			
2.	XL1 Blue	General cloning host; Genotype: endA1 gyrA96(nalR) thi-1 recA1 relA1 lac glnV44 F'[::Tn10 proAB+ lacIq Δ(lacZ)M15] hsdR17(rK- mK+)		TetR			
3.	BL21(DE3)	Expression host, Genotype: F- ompT hsdS(rB-mB-)gal dcm(DE3)		-			
4.	Origami(DE3)	Expression host, Genotype: F- ompT hsdSB(rB- mB-) gal dcm lacY1 ahpC (DE3) gor522:: Tn10 trxB (KanR, TetR)		KanR+TetR			
5.	MG1655	Expression host, Genotype: K-12 F– λ– ilvG– rfb-50 rph-1		-			
6.	AD494	Expression host, Genotype: D (ara-leu)7967, lacX74, phoAPvuII, phoR, malF3, trxB::Kan		KanR			

Supplemental Table 2. List of primers used in this study

Primer name	Sequences
J270	TTTTTTTTCATATGGCGCACTACAACACCGAG
J271	TTTTTTTTGGATCCACGACGAATGATGCTGTGAAC
J273	TTTTTTTTGGATCCGCTCATTTGCGGACG
J286	TTTTTTTTCATATGGCAGCATTCGAGAGCGGCCTGGAC
J289	TTTTTTTTCATATGAGCCGTACCGAGGAAACCATC
J290	TTTTTTTTGGATCCCAGCTTGCTCATGCAACG
M12488	CCGCGAAGACTTCATGACAGAAGAGACTATAAAATTTGC
M18862	CCGGATCCTCAACGTCTAATAATGGAATGAACTTG
NcoI-VEGF-C-Fw	CCGGCCATGGATACAGAAGAGACTATAAAATTTGCTGCAG
M12598	CCGGATCCTCAATCCAGTTTAGACATGCATC
M12597	CCGCGAAGACTTCATGGCGCATTATAATACAGAGATCTTGAAAAG

Supplemental Table 3a. Conditions of the 1st folding screen

	pH	Buffer (mM)	Salt (mM)	PEG 3350 (%)	G: guanidine HCl, U: urea (mM)	Cation/ Chelator (mM)	Polar/ nonpolar additives (mM)	Redox components (mM)	LM: Lauryl maltoside, C: CHAPS (mM)	Remarks (+/++ minor/significant precipitation during dialysis)
**1**	8.2	55 Tris	264 NaCl, 11 KCl	0.055		1.1 EDTA		1 DTT		**+**
**2**	6.5	55 MES	10.56 NaCl, 0.44 KCl		550 G	2.2 $MgCl_2$, 2.2 CaCl		1/0.1 GSH/GSSG	0.3 LM	+
**3**	6.5	55 MES	10.56 NaCl, 0.44 KCl	0.055	550 G	1.1 EDTA	440 sucrose, 550 L-arginine	1/0.1 GSH/GSSG		+
**4**	8.2	55 Tris	264 NaCl, 11 KCl			2.2 $MgCl_2$, 2.2 CaCl	440 sucrose, 550 L-arginine	1 DTT	0.3 LM	+
**5**	6.5	55 MES	264 NaCl, 11 KCl			2.2 $MgCl_2$, 2.2 CaCl	440 sucrose	1/0.1 GSH/GSSG		+
**6**	8.2	55 Tris	10.56 NaCl, 0.44 KCl	0.055	550 G	1.1 EDTA	440 sucrose	1 DTT	0.3 LM	++
**7**	8.2	55 Tris	10.56 NaCl, 0.44 KCl		550 G	2.2 $MgCl_2$, 2.2 CaCl	550 L-arginine	1 DTT		++
**8**	6.5	55 MES	264 NaCl, 11 KCl	0.055		1.1 EDTA	550 L-arginine	1/0.1 GSH/GSSG	0.3 LM	++
**9**	6.5	55 MES	264 NaCl, 11 KCl	0.055	550 G	2.2 $MgCl_2$, 2.2 CaCl	440 sucrose	1 DTT		
**10**	8.2	55 Tris	10.56 NaCl, 0.44 KCl			1.1 EDTA	440 sucrose	1/0.1 GSH/GSSG	0.3 LM	
**11**	8.2	55 Tris	10.56 NaCl, 0.44 KCl	0.055		2.2 $MgCl_2$, 2.2 CaCl	550 L-arginine	1/0.1 GSH/GSSG		
**12**	6.5	55 MES	264 NaCl, 11 KCl		550 G	1.1 EDTA	550 L-arginine	1 DTT	0.3 LM	
**13**	8.2	55 Tris	264 NaCl, 11 KCl		550 G	1.1 EDTA		1/0.1 GSH/GSSG		++
**14**	6.5	55 MES	10.56 NaCl, 0.44 KCl	0.055		2.2 $MgCl_2$, 2.2 CaCl		1 DTT	0.3 LM	++
**15**	6.5	55 MES	10.56 NaCl, 0.44 KCl			1.1 EDTA	440 sucrose, 550 L-arginine	1 DTT		++
**16**	8.2	55 Tris	264 NaCl, 11 KCl	0.055	550 G	2.2 $MgCl_2$, 2.2 CaCl	440 sucrose, 550 L-arginine	1/0.1 GSH/GSSG	0.3 LM	+
**17**	8.5	20 Tris				0.007 $CuCl_2$				
**18**	8.5	20 Tris	0.44 KCl		550 G					
**19**	8.5	20 Tris			550 G					
**20**	8.5	20 Tris						1/1 GSH/GSSG		
**21**	8.5	20 Tris								
**22**	8.5	20 Tris					550 L-arginine			
**23**	8.5	20 Tris					440 sucrose			
**24**	8.5	20 Tris					550 L-arginine	1/1 GSH/GSSG	10 C	
**25**	8.5	100 Tris			550 G	2 EDTA		5/1 cysteine/cystine		(Scrofani et al., 2000)
**26**	7.4	50 Tris			300 U		50 L-glycine	2 DTT, 2 cystamine		(Siemeister et al., 1996) +
**27**	8.5	20 Tris	400 NaCl							(Christinger et al., 1996) ++
**28**	8.5	20 Tris	400 NaCl					2 DTT		(Christinger et al., 1996) ++
**29**	8.5	20 Tris	400 NaCl			0.007 $CuCl_2$		1 cysteine		(Christinger et al., 1996) ++
**30**	8.5	20 Tris	400 NaCl		300 U					(Christinger et al., 1996) ++
**31**	8.5	20 Tris	400 NaCl		300 U			2 DTT		(Christinger et al., 1996) ++

**32**	8.5	20 Tris	400 NaCl		300 U	0.007 $CuCl_2$		1 cysteine		(Christinger et al., 1996) ++

Table 3a. For rows with white background, solubilized inclusion bodies were added at a low concentration (2 mg/ml), while rows with grey background, they were added at a high concentration (20 mg/ml).

Supplemental Table 3b. Conditions of the 2nd folding screen

	pH	Buffer (mM)	Salt (mM)	PEG 3350 (%)	G: guanidine dine HCl, U: urea (mM)	Cation/ Chelator (mM)	Polar/ nonpolar additives (mM)	Redox components (mM)	LM: Lauryl maltoside	Remarks
**1**	8.5	100 Tris	264 NaCl, 11 KCl	0.055	550 G	2 EDTA		500/100/100 cysteine/cystine/DTT		
**2**	8.5	100 Tris	10.56 NaCl, 0.44 KCl		550 G	2 EDTA, 2.2 $MgCl_2$, 2.2 $CaCl_2$		500/100 cysteine/cystine	0.3 LM	
**3**	8.5	100 Tris	10.56 NaCl, 0.44 KCl	0.055	550 G	2 EDTA	440 sucrose, 550 L-arginine	500/100 cysteine/cystine		
**4**	8.5	100 Tris	264 NaCl, 11 KCl		550 G	2 EDTA, 2.2 $MgCl_2$, 2.2 $CaCl_2$	440 sucrose, 550 L-arginine	500/100/100 cysteine/cystine/DTT	0.3 LM	
**5**	8.5	100 Tris	264 NaCl, 11 KCl		550 G	2 EDTA, 2.2 $MgCl_2$, 2.2 $CaCl_2$	440 sucrose	500/100 cysteine/cystine		
**6**	8.5	100 Tris	10.56 NaCl, 0.44 KCl	0.055	550 G	2 EDTA	440 sucrose	500/100/100 cysteine/cystine/DTT	0.3 LM	
**7**	8.5	100 Tris	10.56 NaCl, 0.44 KCl		550 G	2 EDTA, 2.2 $MgCl_2$, 2.2 $CaCl_2$l	550 L-arginine	500/100/100 cysteine/cystine/DTT		
**8**	8.5	100 Tris	264 NaCl, 11 KCl	0.055	550 G	2 EDTA	550 L-arginine	500/100 cysteine/cystine	0.3 LM	
**9**	8.5	100 Tris	264 NaCl, 11 KCl	0.055	550 G	2 EDTA, 2.2 $MgCl_2$, 2.2 $CaCl_2$	440 sucrose	500/100/100 cysteine/cystine/DTT		
**10**	8.5	100 Tris	10.56 NaCl, 0.44 KCl		550 G	2 EDTA	440 sucrose	500/100 cysteine/cystine	0.3 LM	
**11**	8.5	100 Tris	10.56 NaCl, 0.44 KCl	0.055	550 G	2 EDTA, 2.2 $MgCl_2$, 2.2 $CaCl_2$	550 L-arginine	500/100 cysteine/cystine		
**12**	8.5	100 Tris	264 NaCl, 11 KCl		550 G	2 EDTA	550 L-arginine	500/100/100 cysteine/cystine/DTT	0.3 LM	
**13**	8.5	100 Tris	264 NaCl, 11 KCl		550 G	2 EDTA		500/100 cysteine/cystine		
**14**	8.5	100 Tris	10.56 NaCl, 0.44 KCl	0.055	550 G	2 EDTA, 2.2 $MgCl_2$, 2.2 $CaCl_2$		500/100/100 cysteine/cystine/DTT	0.3 LM	
**15**	8.5	100 Tris	10.56 NaCl, 0.44 KCl		550 G	2 EDTA	440 sucrose, 550 L-arginine	500/100/100 cysteine/cystine/DTT		
**16**	8.5	100 Tris	264 NaCl, 11 KCl	0.055	550 G	2 EDTA, 2.2 $MgCl_2$, 2.2 $CaCl_2$	440 sucrose, 550 L-arginine	500/100 cysteine/cystine	0.3 LM	

Supplemental Table 3c. Conditions of the 3rd folding screen

	pH	Buffer (mM)	Salt (mM)	PEG 3350 (%)	G: guanidine dine HCl, U: urea (mM)	Cation/ Chelator (mM)	Polar/ nonpolar additives (mM)	Redox components (mM)	LM: Lauryl maltoside	Remarks
**1**	8.5	100 Tris	264 NaCl, 11 KCl	0.055	550 G	2 EDTA		500/100 cysteine/cystine		
**2**	8.5	100 Tris	10.56 NaCl, 0.44 KCl		550 G	2 EDTA		1/1 GSH/GSSG	0.3 LM	
**3**	8.5	100 Tris	10.56 NaCl, 0.44 KCl	0.055	550 G	2 EDTA	440 sucrose, 550 L-arginine	1/1 GSH/GSSG		
**4**	8.5	100 Tris	264 NaCl, 11 KCl		550 G	2 EDTA	440 sucrose, 550 L-arginine	500/100 cysteine/cystine	0.3 LM	
**5**	8.5	100 Tris	264 NaCl, 11 KCl		550 G	2 EDTA	440 sucrose	1/1 GSH/GSSG		
**6**	8.5	100 Tris	10.56 NaCl, 0.44 KCl	0.055	550 G	2 EDTA	440 sucrose	500/100 cysteine/cystine	0.3 LM	
**7**	8.5	100 Tris	10.56 NaCl, 0.44 KCl		550 G	2 EDTA	550 L-arginine	500/100 cysteine/cystine		
**8**	8.5	100 Tris	264 NaCl, 11 KCl	0.055	550 G	2 EDTA	550 L-arginine	1/1 GSH/GSSG	0.3 LM	
**9**	8.5	100 Tris	264 NaCl, 11 KCl	0.055	550 G	2 EDTA	440 sucrose	500/100 cysteine/cystine		
**10**	8.5	100 Tris	10.56 NaCl, 0.44 KCl		550 G	2 EDTA	440 sucrose	1/1 GSH/GSSG	0.3 LM	
**11**	8.5	100 Tris	10.56 NaCl, 0.44 KCl	0.055	550 G	2 EDTA	550 L-arginine	1/1 GSH/GSSG		
**12**	8.5	100 Tris	264 NaCl, 11 KCl		550 G	2 EDTA	550 L-arginine	500/100 cysteine/cystine	0.3 LM	
**13**	8.5	100 Tris	264 NaCl, 11 KCl		550 G	2 EDTA		1/1 GSH/GSSG		
**14**	8.5	100 Tris	10.56 NaCl, 0.44 KCl	0.055	550 G	2 EDTA		500/100 cysteine/cystine	0.3 LM	
**15**	8.5	100 Tris	10.56 NaCl, 0.44 KCl		550 G	2 EDTA	440 sucrose, 550 L-arginine	500/100 cysteine/cystine		
**16**	8.5	100 Tris	264 NaCl, 11 KCl	0.055	550 G	2 EDTA	440 sucrose, 550 L-arginine	1/1 GSH/GSSG	0.3 LM	
**17**	8.5	100 Tris			550 G	2 EDTA		500/100 cysteine/cystine		
**18**	8.5	100 Tris		0.055	550 G	2 EDTA		500/100 cysteine/cystine		
**19**	8.5	100 Tris			550 G	2 EDTA	440 sucrose	500/100 cysteine/cystine		
**20**	8.5	100 Tris			550 G	2 EDTA	550 L-arginine	500/100 cysteine/cystine		

Supplemental Table 3d. Conditions of the 4th folding screen

	pH	Buffer (mM)	Salt (mM)	PEG 3350 (%)	G: guanidine HCl, U: urea (mM)	Cation/ Chelator (mM)	Polar/ nonpolar additives (mM)	Redox components (mM)	LM: Lauryl maltoside	Remarks
**1**	8.5	100 Tris	264 NaCl, 11 KCl		550 G	2 EDTA	440 sucrose	1/1 GSH/GSSG		
**2**	8.5	100 Tris	10.56 NaCl, 0.44 KCl	0.055	550 G	2 EDTA	550 L-arginine	1/1 GSH/GSSG		
**3**	8.5	100 Tris	264 NaCl, 11 KCl		550 G	2 EDTA		1/1 GSH/GSSG		
**4**	8.5	100 Tris	264 NaCl, 11 KCl		550 G	2 EDTA		5/1 cysteine/cystine		
**5**	8.5	100 Tris			550 G	2 EDTA		5/1 cysteine/cystine		
**6**	8.5	100 Tris	264 NaCl, 11 KCl		550 G	2 EDTA	440 sucrose	1/1 GSH/GSSG		
**7**	8.5	100 Tris	10.56 NaCl, 0.44 KCl	0.055	550 G	2 EDTA	550 L-arginine	1/1 GSH/GSSG		
**8**	8.5	100 Tris	264 NaCl, 11 KCl		550 G	2 EDTA		1/1 GSH/GSSG		
**9**	8.5	100 Tris	264 NaCl, 11 KCl		550 G	2 EDTA		5/1 cysteine/cystine		
**10**	8.5	100 Tris			550 G	2 EDTA		5/1 cysteine/cystine		
**11**	6.5	55 MES	10.56 NaCl, 0.44 KCl		550 G	2.2 $MgCl_2$, 2.2 CaCl		1/0.1 GSH/GSSG	0.3 LM	
**12**	8.2	55 Tris	264 NaCl, 11 KCl		550 G	1.1 EDTA		1/0.1 GSH/GSSG		
**13**	8.5	100 Tris			550 G	2 EDTA		5/1 cysteine/cystine		
**14**	7.4	50 Tris			300 U		50 L-glycine	1/0.1/2.5 GSH/ GSSG/cystamine		
**15**	7.4	50 Tris			300 U		50 L-glycine	5/1/2.5 cysteine/ cystine/cystamine		
**16**	6.5	55 MES	10.56 NaCl, 0.44 KCl		550 G	2.2 $MgCl_2$, 2.2 CaCl		1/0.1 GSH/GSSG	0.3 LM	
**17**	8.2	55 Tris	264 NaCl, 11 KCl		550 G	1.1 EDTA		1/0.1 GSH/GSSG		
**18**	8.5	100 Tris			550 G	2 EDTA		5/1 cysteine/cystine		
**19**	7.4	50 Tris			300 U		50 L-glycine	1/0.1/2.5 GSH/ GSSG/cystamine		
**20**	7.4	50 Tris			300 U		50 L-glycine	5/1/2.5 cysteine/ cystine/cystamine		
