# Supplementary material for: Bioactive VEGF-C from E. coli
Source: Sci Rep. 2022 Oct 28;12:18157. doi: 10.1038/s41598-022-22960-0 (PMC9616921; doi:10.1038/s41598-022-22960-0)
Supplement: Supplementary file 2 — Supplementary Information 2. [file 41598_2022_22960_MOESM2_ESM.pdf]

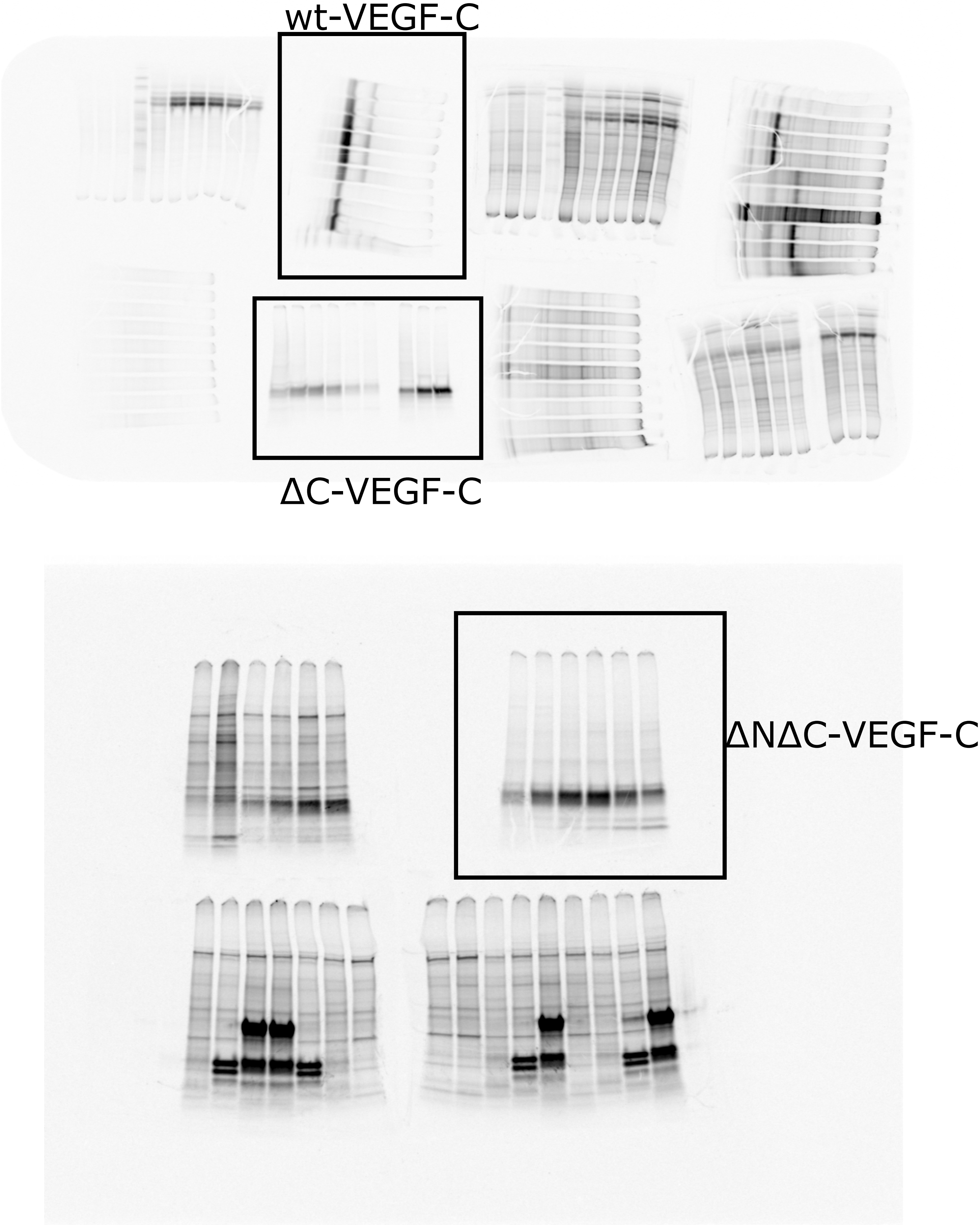

Fig1a. Full-length gels exposed on phosphoimager plates

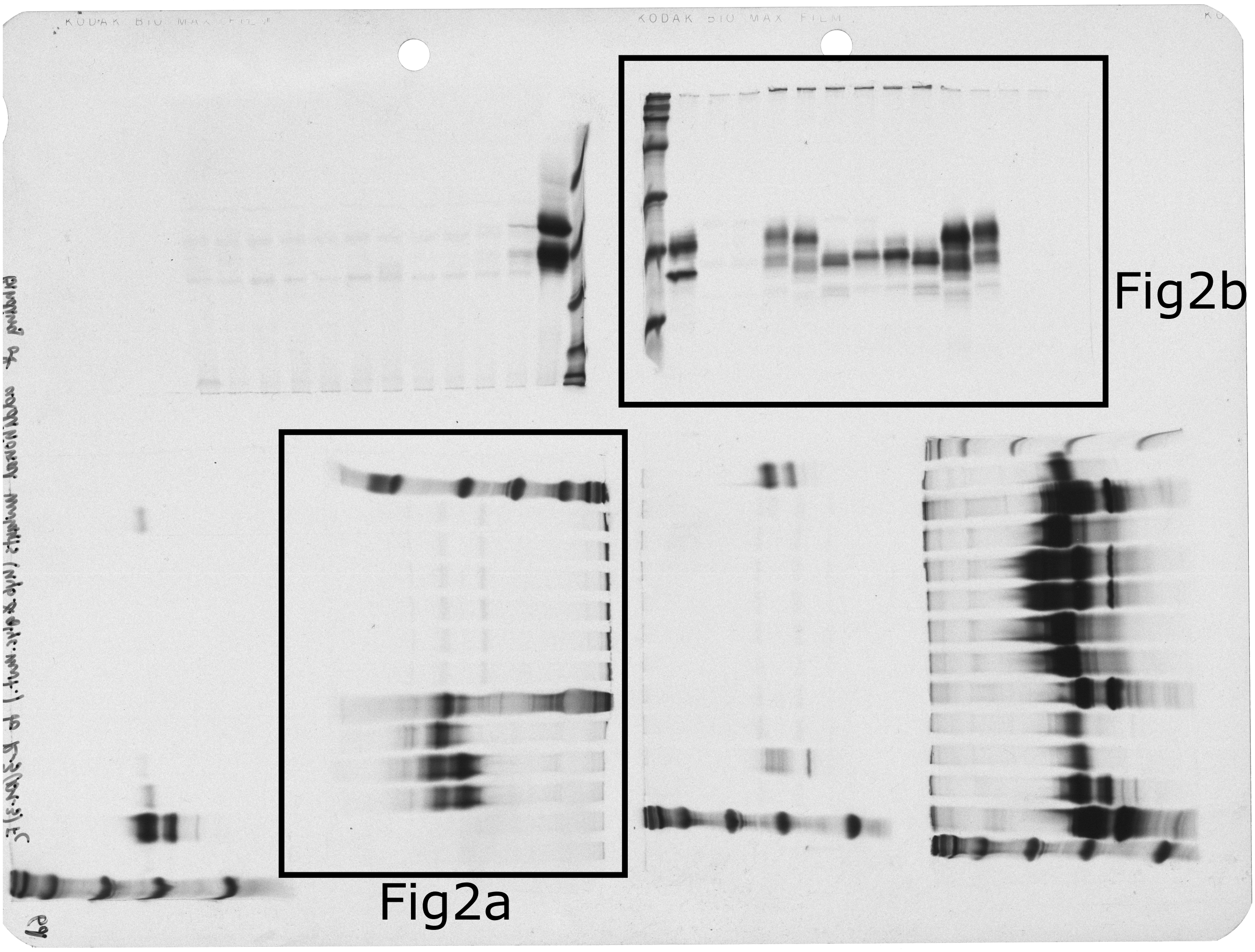

Fig2 (a+b) X-ray film exposure of full-length gels

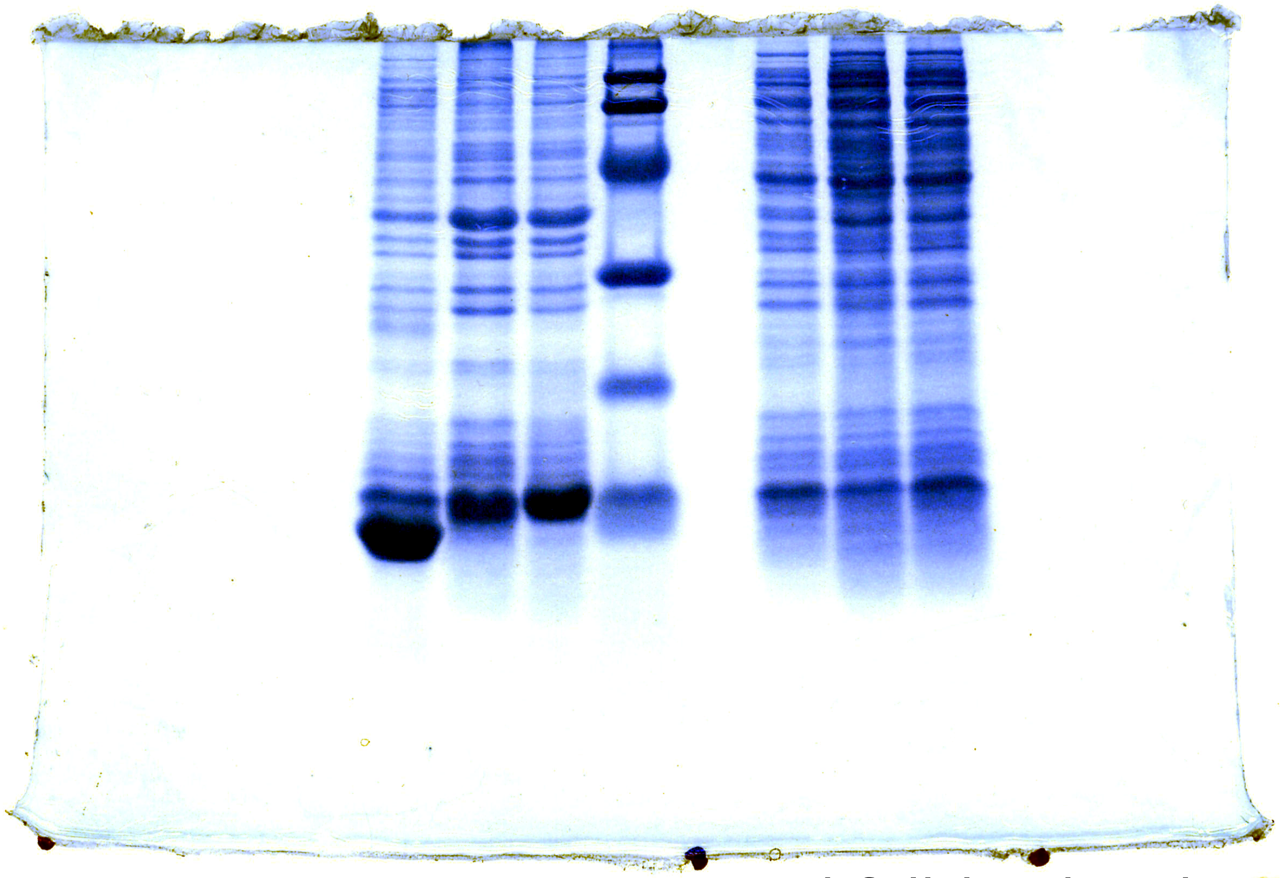

Fig2c Coomassie-stained full-lenth gel

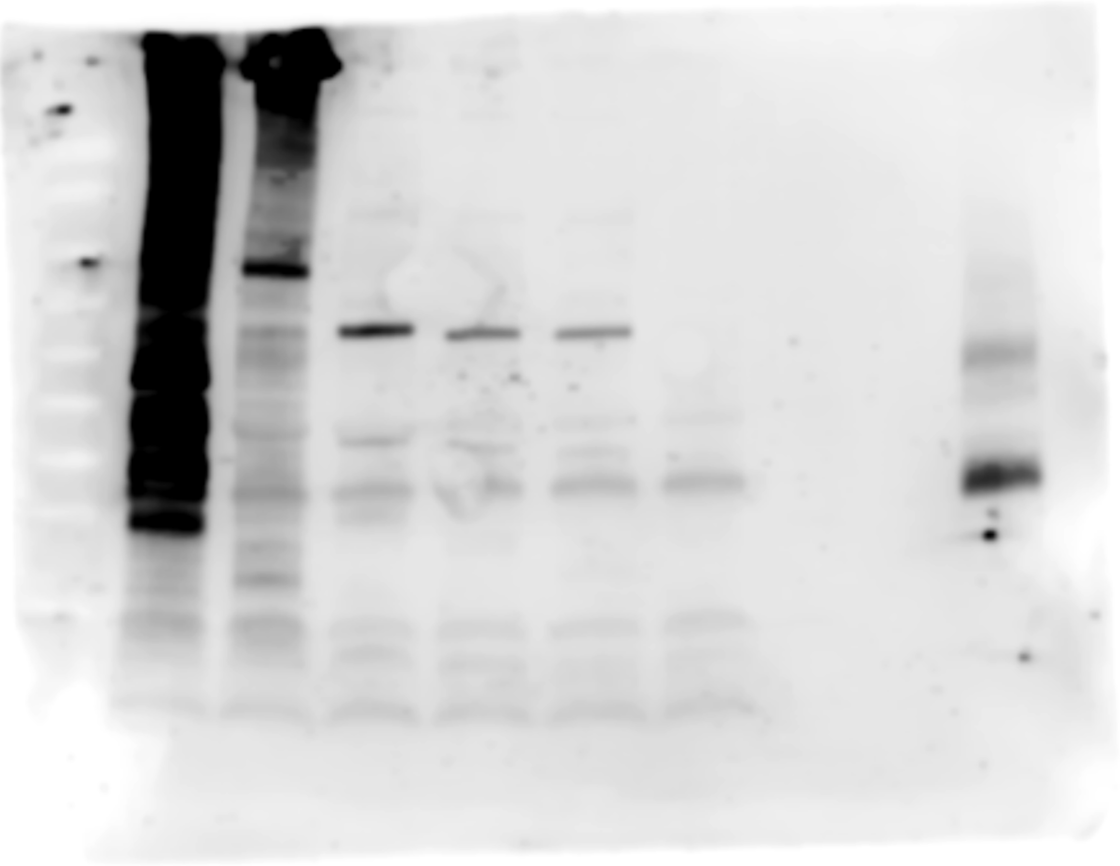

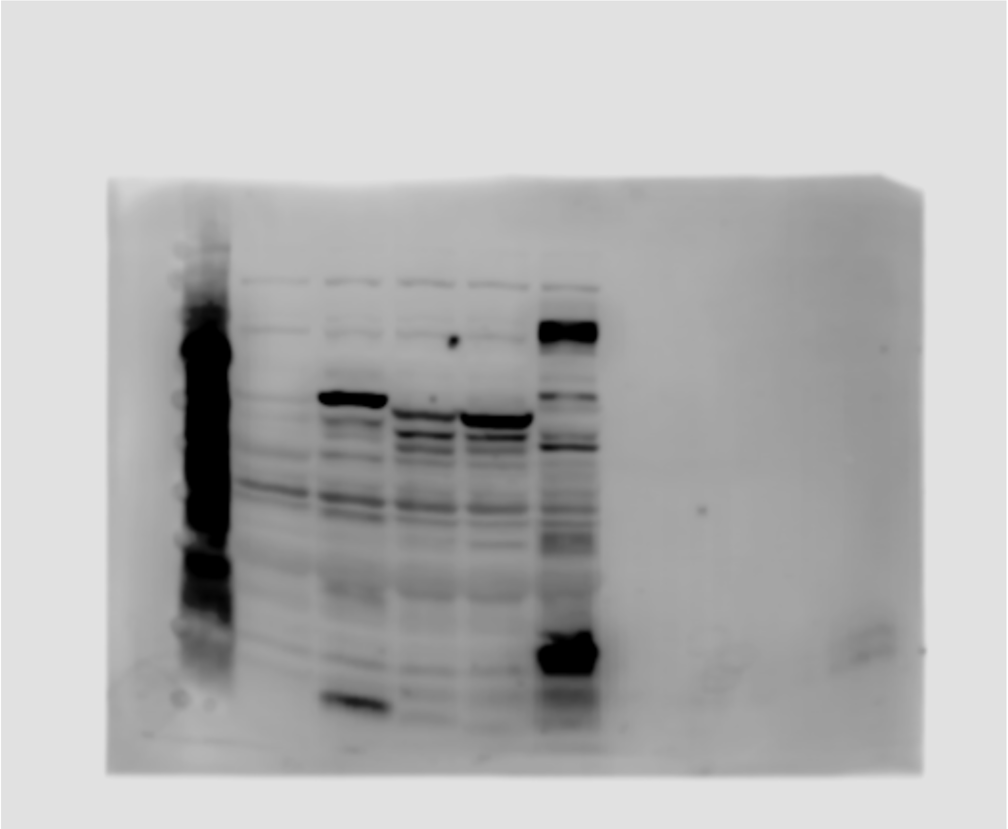

Fig3a and c Full-length western blots

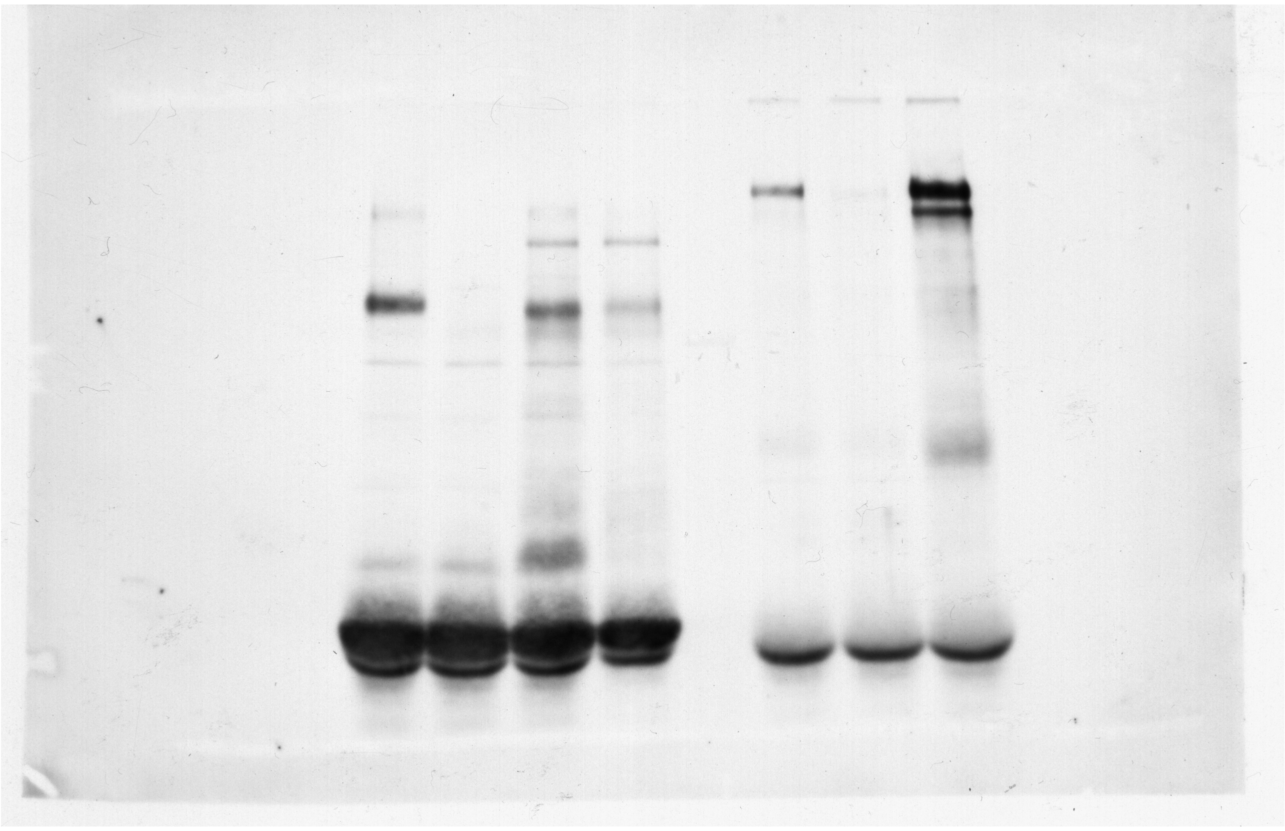

Fig4b Full-length western blots

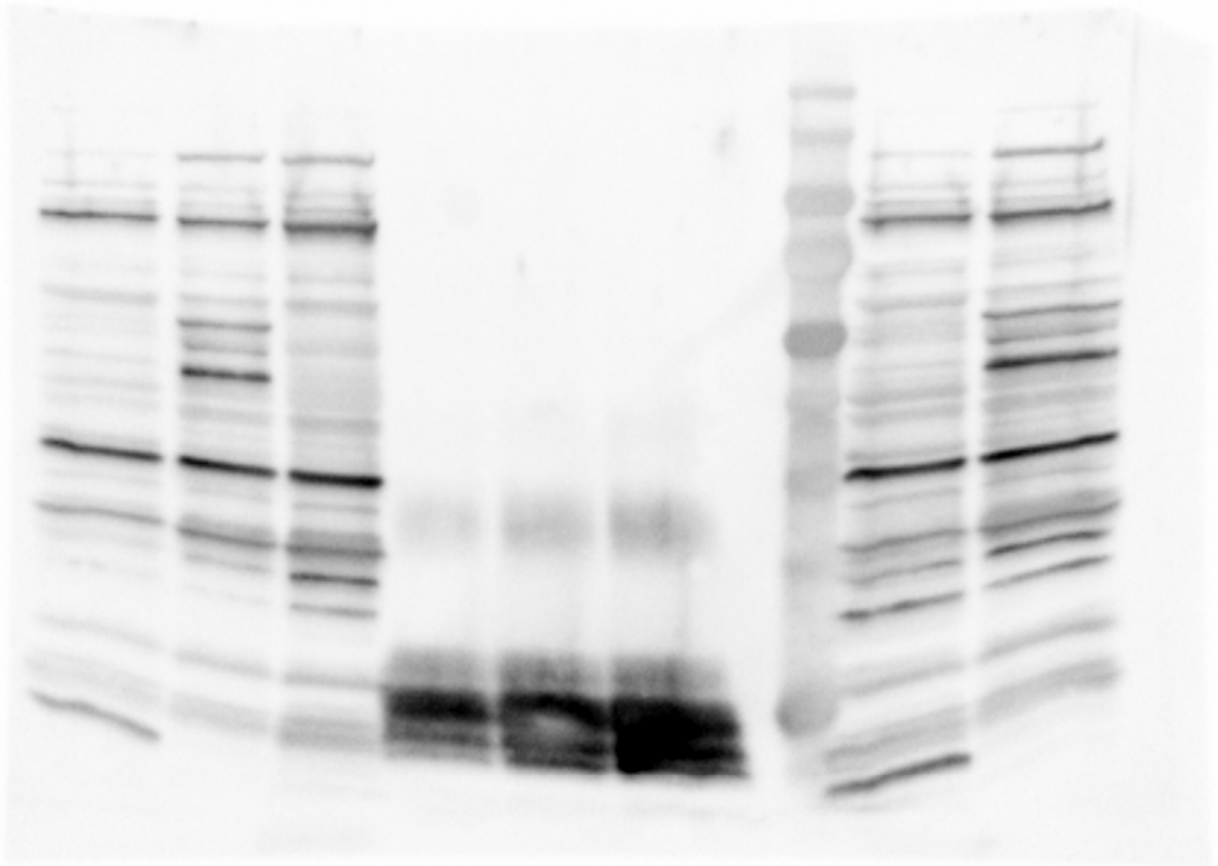

Fig5a Full-length western blots

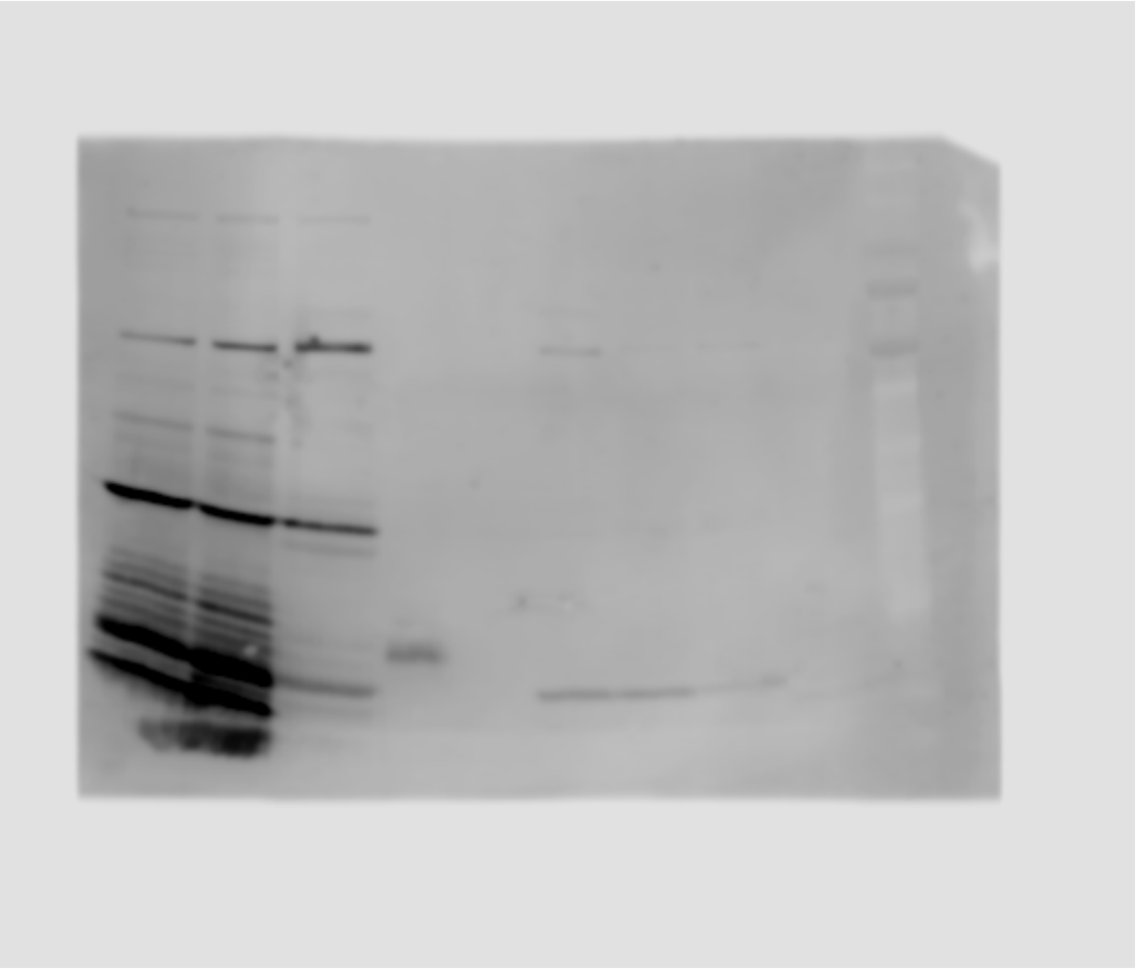

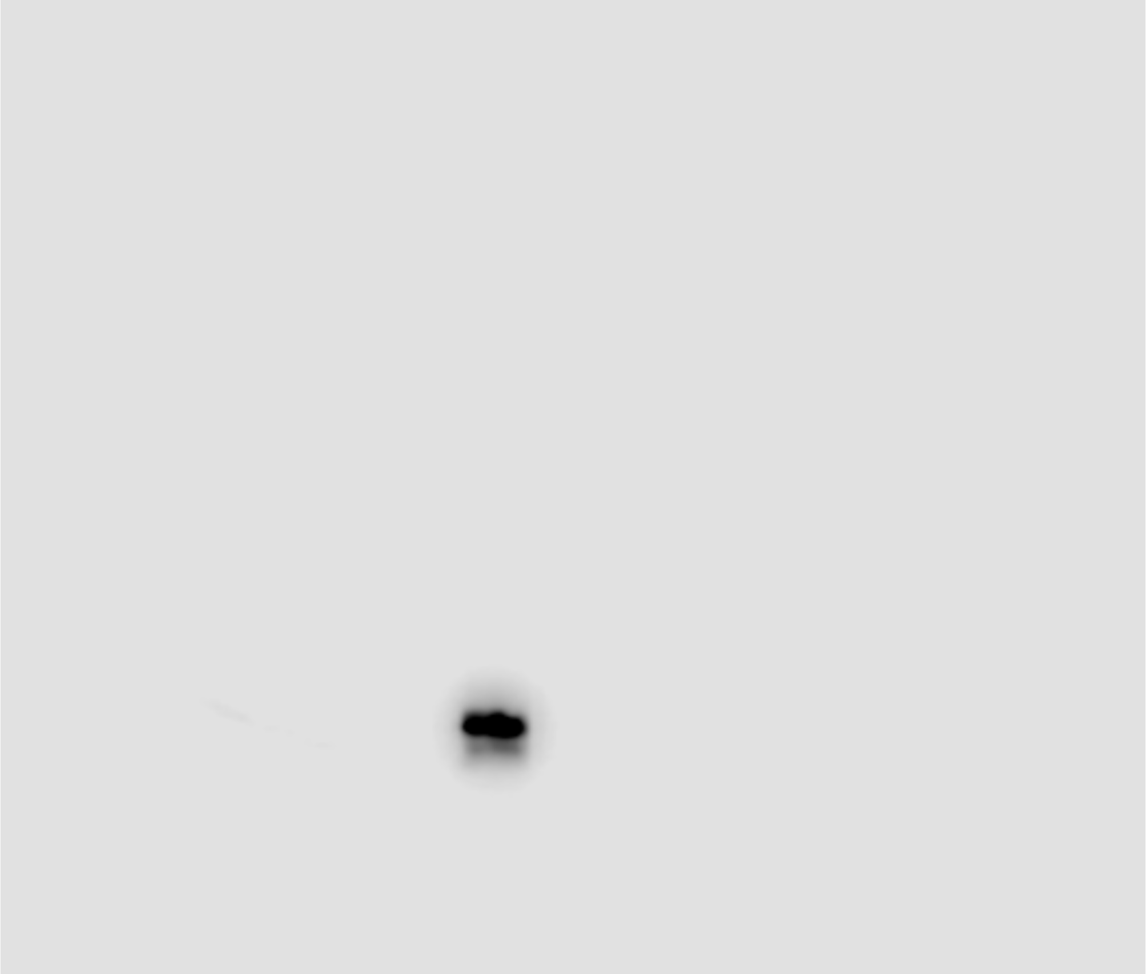

Fig6b and c Full-length western blots

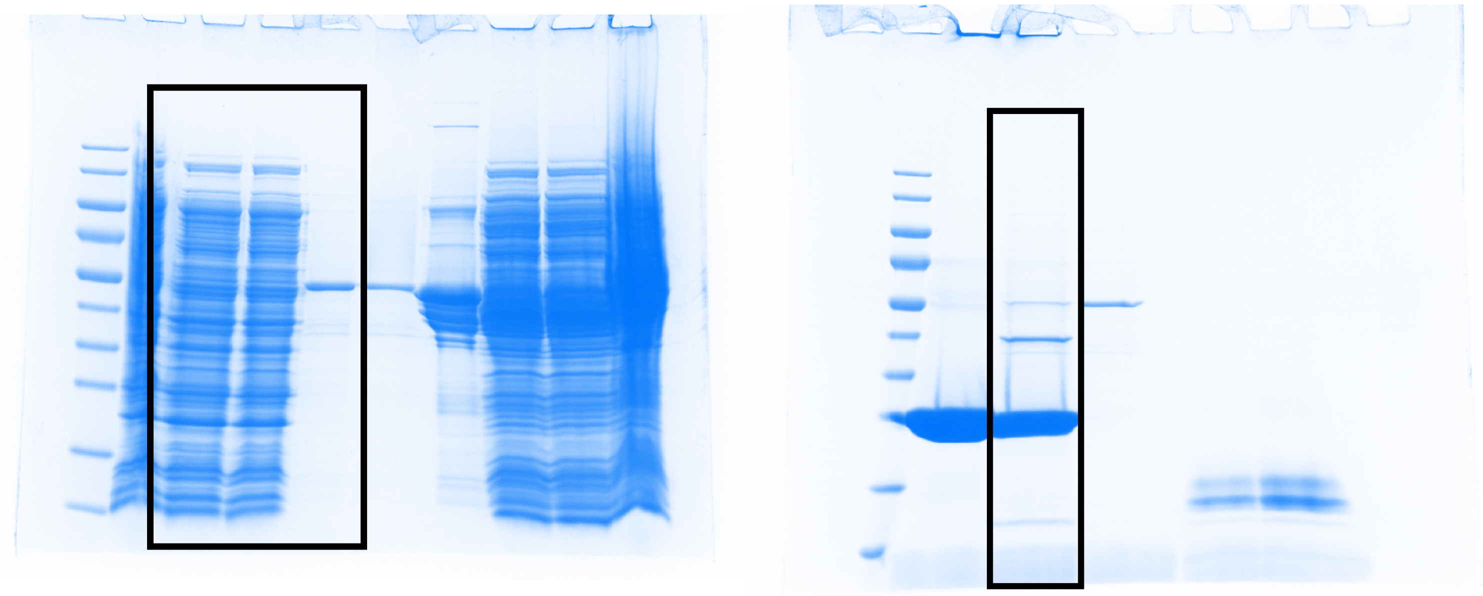

Fig7b Full-length coomassie-stained gels

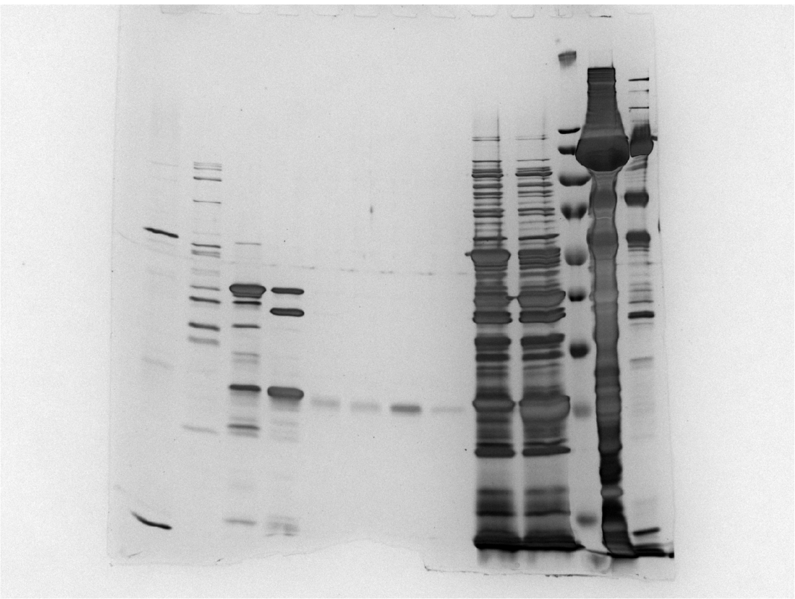

Fig7c Full-length silver-stained gel

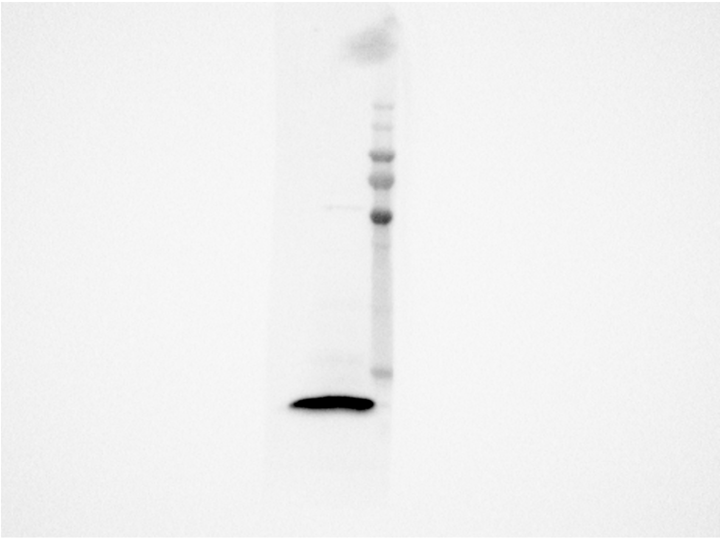

Fig7d Full-length western blot

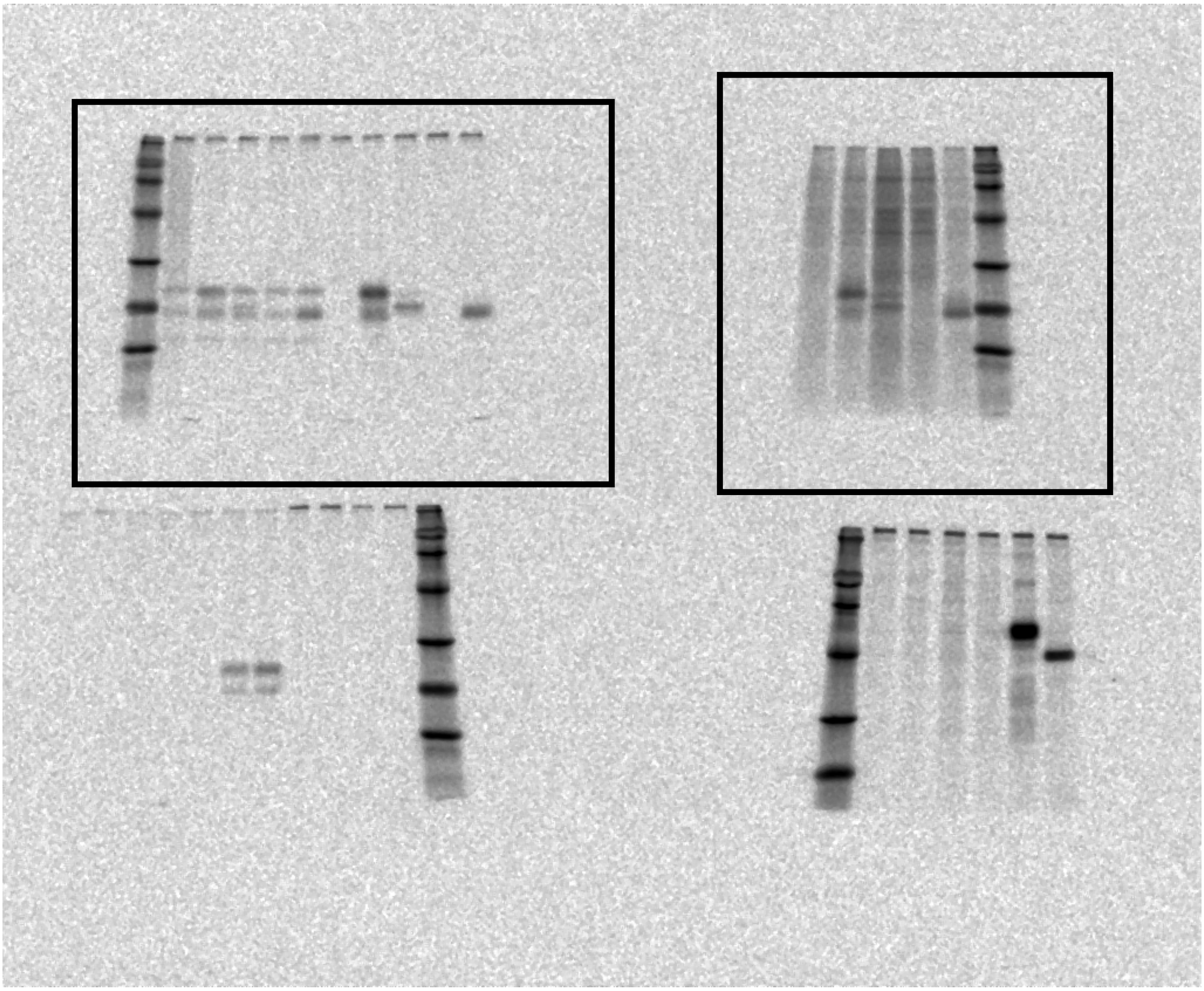

FigS1a and b Full-length gels exposed on phosphoimager plate

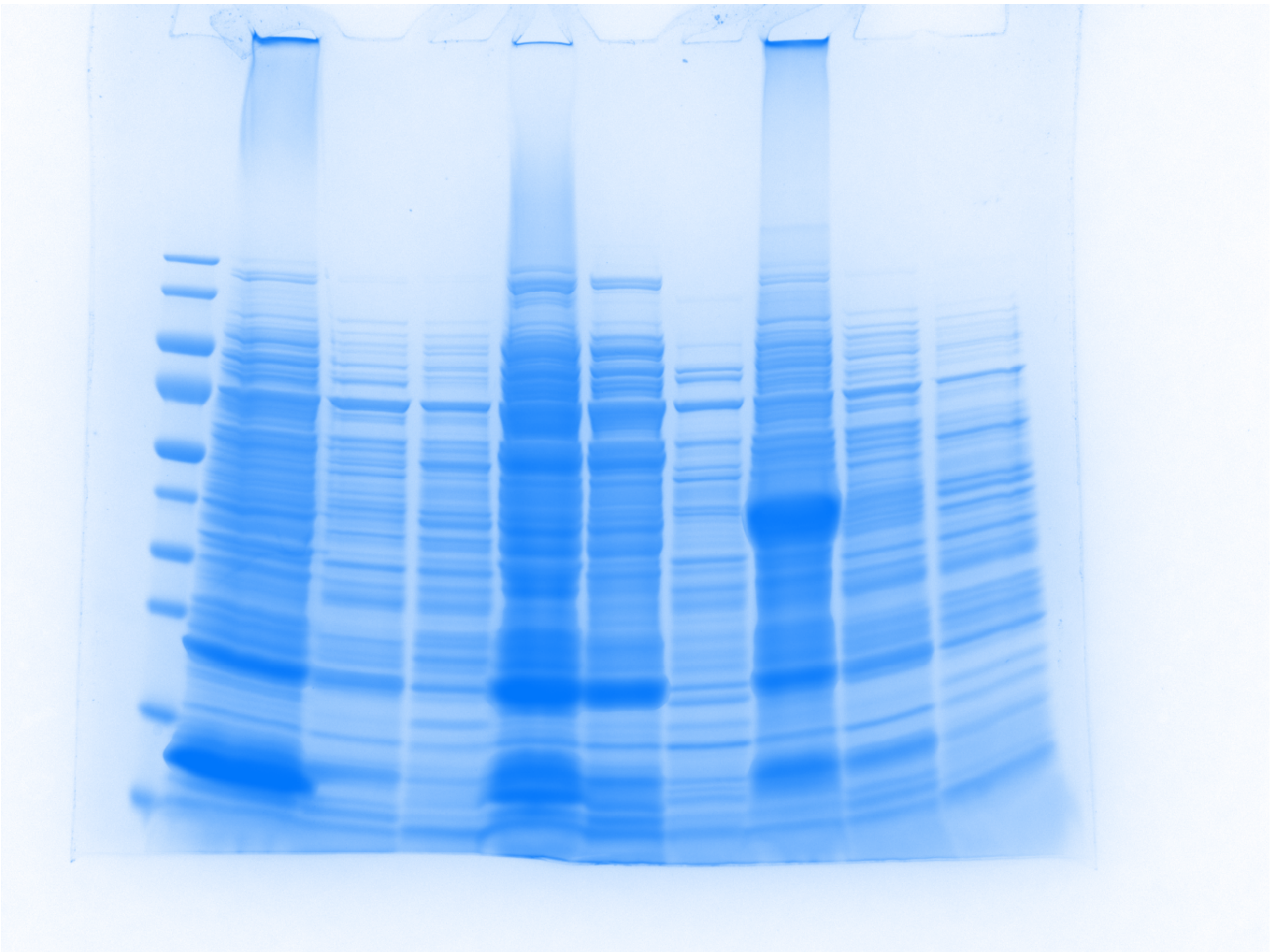

FigS2a Full-length coomassie-stained gel

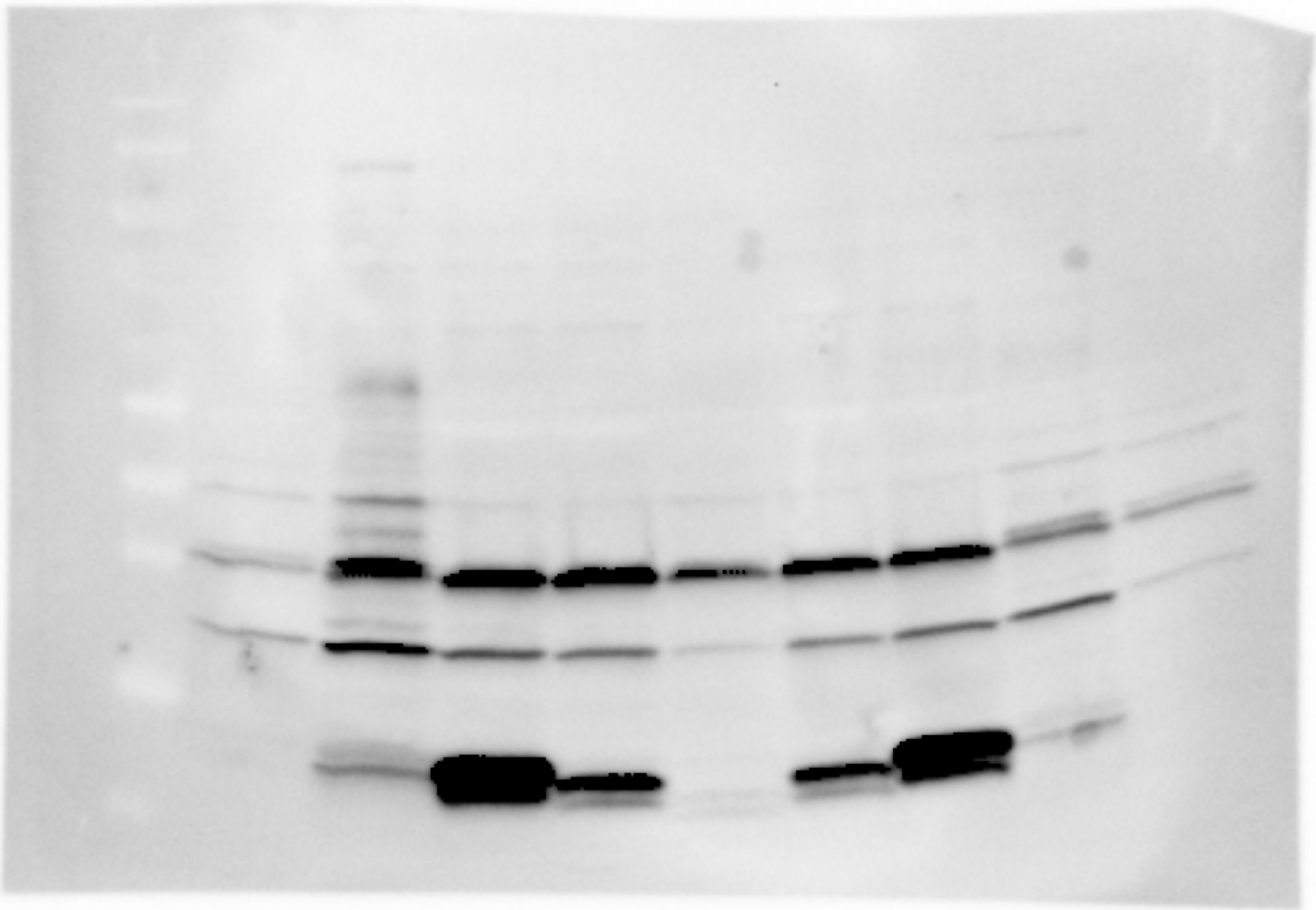

FigS3a Full-length western blot

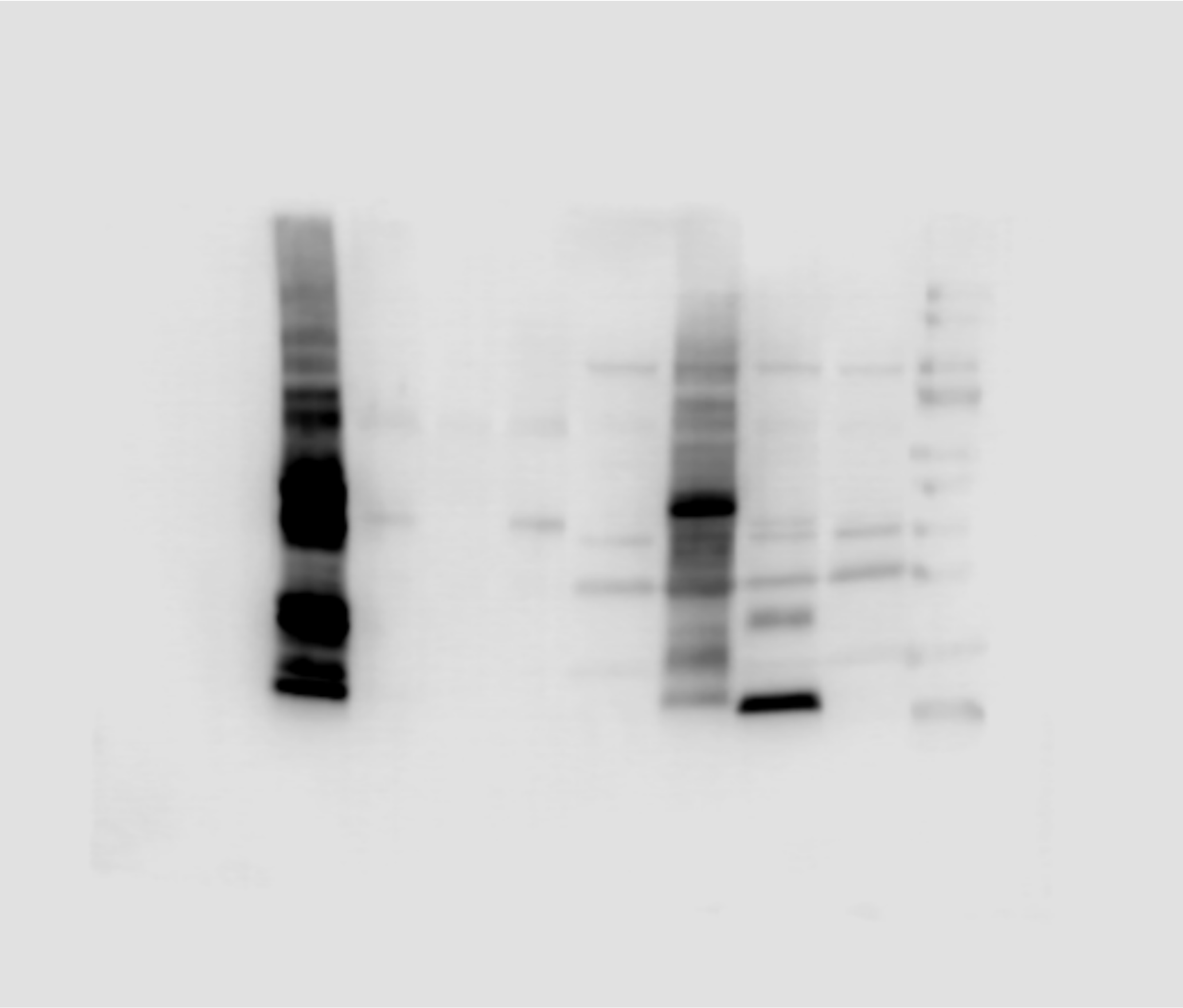

FigS4a Full-length western blot
